# Supplementary material for: Feasibility study of eye movement desensitisation and reprocessing (EMDR) in people with an at-risk mental state (ARMS) for psychosis: study protocol
Source: BMJ Open. 2020 Oct 1;10(10):e038620. doi: 10.1136/bmjopen-2020-038620 (PMC7534702; doi:10.1136/bmjopen-2020-038620)
Supplement: Author's manuscript [file bmjopen-2020-038620.draft_revisions.pdf]

BMJ Open is committed to open peer review. As part of this commitment we make the peer review history of every article we publish publicly available.

When an article is published we post the peer reviewers' comments and the authors' responses online. We also post the versions of the paper that were used during peer review. These are the versions that the peer review comments apply to.

The versions of the paper that follow are the versions that were submitted during the peer review process. They are not the versions of record or the final published versions. They should not be cited or distributed as the published version of this manuscript.

BMJ Open is an open access journal and the full, final, typeset and author-corrected version of record of the manuscript is available on our site with no access controls, subscription charges or pay-per-view fees (http://bmjopen.bmj.com).

If you have any questions on BMJ Open's open peer review process please email info.bmjopen@bmj.com

BMJ Open

A feasibility study of eye-movement desensitization and reprocessing (EMDR) in people with an at-risk mental state (ARMS) for psychosis: study protocol

Journal:	*BMJ Open*
Manuscript ID	bmjopen-2020-038620
Article Type:	Protocol
Date Submitted by the Author:	18-Mar-2020
Complete List of Authors:	Strelchuk, Daniela; University of Bristol, Centre for Academic Mental Health, Population Health Sciences, Bristol Medical School; University Hospitals Bristol NHS Foundation Trust, National Institute for Health Research Bristol Biomedical Research Centre<br>Wiles, Nicola; University of Bristol, Centre for Academic Mental Health, Population Health Sciences, Bristol Medical School; University Hospitals Bristol NHS Foundation Trust, National Institute for Health Research Bristol Biomedical Research Centre<br>Turner, Katrina; University of Bristol, Centre for Academic Mental Health, Population Health Sciences, Bristol Medical School; University Hospitals Bristol NHS Foundation Trust, National Institute for Health Research Bristol Biomedical Research Centre<br>Derrick, Catherine; University of Bristol, Centre for Academic Mental Health, Population Health Sciences, Bristol Medical School<br>Zammit, Stan; University of Bristol, Centre for Academic Mental Health, Population Health Sciences, Bristol Medical School; Cardiff University, MRC Centre for Neuropsychiatric Genetics and Genomics, Division of Psychological Medicine and Clinical Neuroscience
Keywords:	Schizophrenia & psychotic disorders < PSYCHIATRY, QUALITATIVE RESEARCH, Clinical trials < THERAPEUTICS

SCHOLARONE™
Manuscripts

I, the Submitting Author has the right to grant and does grant on behalf of all authors of the Work (as defined in the below author licence), an exclusive licence and/or a non-exclusive licence for contributions from authors who are: i) UK Crown employees; ii) where BMJ has agreed a CC-BY licence shall apply, and/or iii) in accordance with the terms applicable for US Federal Government officers or employees acting as part of their official duties; on a worldwide, perpetual, irrevocable, royalty-free basis to BMJ Publishing Group Ltd ("BMJ") its licensees and where the relevant Journal is co-owned by BMJ to the co-owners of the Journal, to publish the Work in this journal and any other BMJ products and to exploit all rights, as set out in our licence.

The Submitting Author accepts and understands that any supply made under these terms is made by BMJ to the Submitting Author unless you are acting as an employee on behalf of your employer or a postgraduate student of an affiliated institution which is paying any applicable article publishing charge ("APC") for Open Access articles. Where the Submitting Author wishes to make the Work available on an Open Access basis (and intends to pay the relevant APC), the terms of reuse of such Open Access shall be governed by a Creative Commons licence – details of these licences and which Creative Commons licence will apply to this Work are set out in our licence referred to above.

Other than as permitted in any relevant BMJ Author's Self Archiving Policies, I confirm this Work has not been accepted for publication elsewhere, is not being considered for publication elsewhere and does not duplicate material already published. I confirm all authors consent to publication of this Work and authorise the granting of this licence.

For peer review only

A feasibility study of eye-movement desensitization and reprocessing (EMDR) in people with an at-risk mental state (ARMS) for psychosis: study protocol

Daniela Strelchuk, MSc, Centre for Academic Mental Health, Population Health Sciences, Bristol Medical School, University of Bristol, Bristol, UK and National Institute for Health Research Bristol Biomedical Research Centre, University Hospitals Bristol NHS Foundation Trust and University of Bristol.

Nicola Wiles, PhD, Centre for Academic Mental Health, Population Health Sciences, Bristol Medical School, University of Bristol, Bristol, UK, and National Institute for Health Research Bristol Biomedical Research Centre, University Hospitals Bristol NHS Foundation Trust and University of Bristol.

Katrina Turner, PhD, Centre for Academic Mental Health, Population Health Sciences, Bristol Medical School, University of Bristol, Bristol, UK, and National Institute for Health Research Bristol Biomedical Research Centre, University Hospitals Bristol NHS Foundation Trust and University of Bristol.

Catherine Derrick, Centre for Academic Mental Health, Population Health Sciences, Bristol Medical School, University of Bristol, Bristol, UK

Stan Zammit, PhD, Centre for Academic Mental Health, Population Health Sciences, Bristol Medical School, University of Bristol, Bristol, UK; MRC Centre for Neuropsychiatric Genetics and Genomics, Division of Psychological Medicine and Clinical Neuroscience, Cardiff University, Cardiff, UK and National Institute for Health Research Bristol Biomedical Research Centre, University Hospitals Bristol NHS Foundation Trust and University of Bristol.

Corresponding author:

Daniela Strelchuk

Oakfield House, Oakfield Grove, Bristol, BS8 2BN;

Email: daniela.strelchuk@bristol.ac.uk

Tel: 07575540193

Word count: 3,990

For peer review only - http://bmjopen.bmj.com/site/about/guidelines.xhtml

Abstract

Introduction: Trauma can play an important role in the development of psychosis, yet no studies have investigated whether a trauma-focused psychological therapy could prevent the onset of psychosis in people at high risk of developing this condition. This study aims to establish whether it would be feasible to conduct a multi-centre randomised controlled trial (RCT) to investigate the clinical and cost effectiveness of eye movement desensitization and reprocessing therapy (EMDR) to prevent the onset of psychosis in people with an at-risk mental state (ARMS).

Methods/analysis: This is a single arm trial with a nested qualitative study where all participants (target n=20) will be offered EMDR. Eligible participants are those who meet criteria for ARMS; have experienced a traumatic event before the onset of ARMS symptomatology; and have at least one symptom of post-traumatic stress disorder (PTSD). Participants will be followed-up at 4, 8 and 12 months after the baseline assessment. The primary outcome measure is transition to psychosis, and secondary outcome measures include severity of psychotic symptoms, PTSD, depression, anxiety, impaired functioning, health status, and resource use. The analysis will aim to establish the rates of recruitment and retention for a large-scale RCT. Interviews with therapists and patients will explore their views of the study and their experiences of delivering or receiving EMDR.

Ethics and dissemination

This protocol has been approved by the South West-Cornwall & Plymouth Research Ethics Committee (Reference 18/SW/0037). Findings will be disseminated through journal publications, conference presentations and meetings with service users, their families, mental health professionals and commissioners.

Trial registration: ISRCTN31976295

Strengths and limitations of the study

- This is the first study to investigate the feasibility of conducting a large multi-centre RCT of EMDR for the prevention of psychosis in people with ARMS
- The study includes qualitative work to explore therapists' and patients' views of the treatment offered/received
- Therapy sessions follow an EMDR protocol specifically designed for people with psychosis
- The decision to change the study design to a single arm trial may limit our ability to draw robust conclusions about recruiting to an RCT

For peer review only - http://bmjopen.bmj.com/site/about/guidelines.xhtml

For peer review only

INTRODUCTION

Psychotic illnesses are one of the leading causes of disability worldwide.[1] The onset of psychosis is often preceded by a prodromal phase, known as an at-risk mental state (ARMS) for psychosis, which is characterized by a series of non-specific or attenuated psychotic symptoms.[2] Approximately 22% of ARMS individuals will transition to psychosis within one year and 36% of them within 3 years.[3]

Various interventions have been used for the prevention of psychosis in those with ARMS, but there is still limited evidence on the effectiveness of these different approaches. Clinical guidelines currently recommend cognitive-behavioural therapy (CBT) as a first-line treatment.[4] However, whilst a recent meta-analysis of randomized controlled trials showed that CBT has a moderate effect on transition to psychosis at 12 months follow-up (RR 0.64, 95% CI 0.44, 0.93), the evidence was of low to moderate quality and the effect was not maintained over the longer-term (18 months: RR 0.55, 95% CI 0.25, 1.19).[5]

Meta-analyses looking at environmental risk factors for psychosis have shown that exposure to trauma is 2-3 times more common in people with ARMS or psychosis than in controls,[6–8] and post-traumatic stress disorder (PTSD) is also more common, although not usually detected.[9] Despite strong evidence showing that trauma is a key factor in the development of psychosis,[10–12] no studies have yet investigated whether a trauma-focused therapy could prevent the onset of psychosis in people at high risk.

Eye movement desensitisation and reprocessing (EMDR) is a trauma-focused therapy that has comparable efficacy to trauma-focused CBT (TF-CBT) in treating PTSD.[13–16] Despite their comparable efficacy, the two forms of therapy are different. While TF-CBT seeks to directly identify and restructure negative beliefs about the self and the consequences of trauma, EMDR does this indirectly, relying more on the integration of emotional, cognitive, sensory, physical, and contextual information to allow adaptive processing of traumatic memories. Unlike TF-CBT, EMDR does not require patients to give a detailed description of the trauma, which they may find distressing. Also, unlike TF-CBT, EMDR does not involve homework, which may make it more suitable for people with a disorganized lifestyle, such as those with ARMS. People with depression have described homework as the most challenging aspect of CBT because it triggers negative memories of school homework and brings challenges with regards to discrete completion of homework tasks.[17] Studies in ARMS population also show that some patients do not like the homework and the structure of CBT,[18] and that up to 50% of people with psychosis do not engage with CBT in clinical services.[19]

For peer review only - http://bmjopen.bmj.com/site/about/guidelines.xhtml

To date, there has been only one randomised controlled trial (RCT) comparing EMDR with treatment as usual in patients with co-morbid psychosis and PTSD. This study showed that EMDR improved PTSD (the primary outcome), and also reduced paranoid delusions, but did not reduce auditory hallucinations at 6 months follow-up.[20] However, the EMDR protocol used in this RCT did not specifically target psychotic symptoms. Furthermore, most participants had chronic forms of psychosis and hence psychotic symptoms might have been less amenable to treatment than more recent onset symptoms present in ARMS subjects.

Objectives

The overarching aim of this study is to establish whether it would be feasible to conduct a large multi-centre RCT to evaluate the clinical and cost-effectiveness of EMDR to prevent the onset of psychosis in people with an at-risk mental state. The study has the following objectives:

1. To estimate the rate of recruitment and retention to inform the large-scale RCT
2. To refine the eligibility criteria, screening and recruitment procedures
3. To optimize the EMDR protocols and learn about the factors which affect the implementation of EMDR
4. To explore patients' and therapists' views of EMDR as a treatment for ARMS
5. To investigate patients' and therapists' views of the study design and study materials
6. To understand what treatment as usual (TAU) consists of for patients with ARMS

METHODS AND ANALYSIS

Study design

The study consists of a single arm feasibility trial with a nested qualitative study, and is conducted in collaboration with the Early Intervention (EI) teams from the Avon and Wiltshire Mental Health Partnership NHS Trust (AWP). All participants will be offered EMDR, and will be assessed at baseline, 4, 8 and 12 months follow-up as described below.

The study design outlined is the final version of the trial protocol. The original protocol was changed as a result of low recruitment. Recruitment challenges are described later.

For peer review only

Inclusion and exclusion criteria

Inclusion criteria

- Aged 16 years or over who are at risk of psychosis (as defined in the Comprehensive Assessment of At-Risk Mental States (CAARMS))[2]
- Presence of at least one positive symptom of psychosis (perceptual abnormality, unusual thought content, non-bizarre ideas or disorganised speech) scored ≥3 on CAARMS
- History of traumatic experience as defined in ICD-10 F43.1, occurring prior to onset of first positive symptom
- Presence of one or more symptoms of re-living, avoidance, hyper-arousal, or cognitive distortions in relation to the traumatic experience (assessed using the PTSD Checklist for DSM-V (PCL5)) during the last month.[21]

Exclusion criteria

- History of treated or untreated psychotic illness or learning disability
- Currently taking antipsychotics
- Currently receiving psychological therapy
- Completed a trauma-focused psychological therapy in the last 2 years
- Insufficient fluency in English
- Lacking mental capacity to provide valid informed consent

Recruitment procedure

Participants will be identified via two routes:

1. Early Intervention services

Researchers will identify clinical teams who are willing to identify potential participants via the following routes:

a) during routine clinic appointments - clinicians will introduce the study to patients who have met ARMS criteria within the past 12 months. Interested patients will be asked to complete section 1 of an Expression of Interest (EOI) form, and to provide their contact details. Completed EOI forms will be returned to the research team by secure email. A member of the research team will telephone interested individuals and, if the individual is willing to take part, agree a time and place for the researcher to meet with them to complete the eligibility assessment. Following the telephone call, the researcher will post the individual a full participant information sheet and a letter confirming the appointment.

Individuals who do not want to hear more about the study will be asked to complete section 2 of the EOI form, which asks for information on age, gender, reasons for non-participation and willingness to take part in a short interview (see 'qualitative data collection' for more detail).

For peer review only - http://bmjopen.bmj.com/site/about/guidelines.xhtml

b) postal or telephone invitations from clinicians – If clinicians do not introduce the study to patients during the appointment, they will post ARMS patients a brief information sheet about the study, an EOI form and a stamped addressed envelope. Clinicians may also introduce the study to patients over the telephone. If the patient is interested, the clinician will ask the patient for permission to share their contact details with the research team. The clinician will then fill in the “Phone permission to contact” form and will email it to the research team. Recruitment will then proceed as outlined in (a).

c) postal invitations sent out by NIHR CRN Clinical Studies Officers (CSO) - CSOs will pre-screen potential participants using the electronic patient record system (RIO), and liaise with the care team regarding potentially eligible patients who will then be sent a brief information sheet and details on how to contact the research team if they are interested in the study. Patients who do not reply will be followed up by telephone by the CSO within a week of receiving the documents. If the patient is interested in the study, the CSO will take permission over the phone to share their contact details with the research team. Once the research team have received contact details, recruitment will proceed as outlined in (a).

2. Everyone included within AWP

‘Everyone Included’ is a standard approach to research in AWP, whereby service users are routinely informed about relevant research opportunities. The AWP Research and Development department will send a ‘Research Opportunity Letter’ on behalf of the research team to potential participants briefly explaining the study and what it involves. A researcher will telephone individuals who are interested in the study and answer any questions. If the individual is willing to take part, an eligibility assessment will be arranged.

Baseline eligibility assessment

Individuals interested in taking part will be invited to a face-to-face appointment with a researcher to establish eligibility, answer questions about participation, and obtain written informed consent.

In order to establish eligibility, patients will be asked to complete the following scales:

- Life-events Checklist for DSM-5[22]
- Childhood Trauma Questionnaire[23]
- PTSD Checklist for DSM-5 (PCL5)[21]

For peer review only

Those who are eligible will be asked to provide written informed consent for their participation in the trial and sociodemographic information. Additional baseline data will be collected using the following quantitative measures:

- Severity of psychotic symptoms: CAARMS,[2] the Negative Scale of the PANSS,[24] the PSYRAT[25] and the CAPE-42.[26]
- Severity of depression and anxiety: Patient Health questionnaire (PHQ-9) for depression[27] and Generalized Anxiety Disorder Questionnaire (GAD-7) for anxiety[28]
- Impaired functioning: Work and Social Adjustment Scale (WSAS)[29]
- Health status: EQ-5D-5L[30]
- Drug use: The Drug Abuse Screening Test (DAST 10)[31]

Data will also be collected on medication use via self-report measures. The baseline eligibility assessment will last about 90 minutes.

Intervention: EMDR

Participants will receive up to 12 sessions of manualized, weekly, face-to-face EMDR therapy. Treatment has been manualized by EMDR consultants from Lancashire and South Cumbria Care NHS Foundation Trust (LSCFT) and individualised to target psychotic symptoms. The treatment protocol was developed and evaluated through pilot cases with first episode psychosis clients,[32] and further refined as part of an ongoing feasibility randomised controlled trial funded by the National Institute of Health Research (PB-PG-0317-20037; ISRCTN43816889). Therapeutic sessions will be delivered by EMDR UK & Europe trained therapists who will receive training based on the LSCFT manual for the study. The training will be tailored to therapists' needs, and will last between 4 to 8 hours. All EMDR therapists will have at least one year of experience of providing EMDR, and monthly supervision with an EMDR consultant will be in place. Each session will last approximately 90 minutes. The first 2-4 sessions will focus on establishing the therapeutic alliance, preparation for EMDR and stabilization techniques, assessment and identification of targets, cognitions, emotions and bodily sensations. The following 8-10 sessions will focus on desensitisation, installation of positive cognitions and body scan for past, present and future stressful situations.

Written permission will be sought to audio-record the therapeutic sessions. 10% of the sessions will be randomly sampled and evaluated by accredited EMDR therapists independent of the study, to check treatment fidelity, using the EMDR fidelity checklist.[33] Therapists will be encouraged to liaise with participants' care coordinators as this could

For peer review only - http://bmjopen.bmj.com/site/about/guidelines.xhtml

improve participants' adherence to therapy. If a serious adverse event (SAE) occurs in relation to therapy, or participant has difficulties engaging with therapy to the point where therapy is no longer worthwhile, the therapy will be discontinued.

Challenges to recruitment

The original feasibility study was designed as a RCT comparing EMDR with treatment as usual (TAU). TAU in AWP is quite heterogeneous and consists of discharge back to the referrer, signposting to other services, CBT, family intervention or psycho-education. Randomisation took place using a remote computerised randomisation service administered by the Bristol Trials Centre ensuring that allocations were concealed from the recruiting researcher. Randomisation was minimized by psychotic symptom severity (score on positive symptoms on CAARMS <11 or ≥11). However, following challenges to recruitment (described below), the study design was changed to a single arm study.

During the first seven months of the study (May to December 2018), we only recruited three participants (against a target of 21). This poor recruitment was primarily because the number of patients identified as ARMS by the EI teams within AWP was much lower than expected, and only three of the six EI teams we originally planned to recruit from were able to support the study due to financial constraints to managing ARMS clients. Therefore, from July 2019, all participants recruited to the study were offered EMDR. Figure 1 shows the flow of participants in the randomised and single arm phases of the trial.

Sample size and feasibility of recruitment

In the original protocol we had planned to recruit up to 40 participants. The initial sample size was informed by literature guidelines[34] and pragmatic considerations concerning potential difficulties accessing this population. The feasibility study would not be powered to detect important clinical differences between EMDR and TAU groups but would provide estimates of the completion and retention rates that would further assist in planning the recruitment for a future RCT.

Given the challenges with recruitment and modification of our study design (from a randomised to single arm trial), we decreased our target sample size from 40 to 20 participants. This sample size will provide us with reasonable precision around estimates of percentages completing the intervention and follow-ups. For example, if 70% of those who started EMDR complete the intervention, the 95% confidence intervals would be 45% and 88%. Similarly, if 75% of those who started EMDR are followed up to 12 months, the 95% confidence intervals for the retention rate would be 50% and 91%.

For peer review only - http://bmjopen.bmj.com/site/about/guidelines.xhtml

Outcome assessment

The follow-up schedule will involve face-to-face assessments at 4, 8 and 12 months after the baseline assessment. Each follow-up assessment will last about 60 minutes. The primary outcome will be assessed at 12 months follow-up, and the secondary outcomes at 4, 8 and 12 months.

Primary outcome

- Transition to psychosis: ICD-10 diagnosis of psychotic disorder from clinical records or via the CAARMS

Secondary outcomes

- Severity of psychotic symptoms: CAARMS, PSYRAT, the negative scale of the PANSS and CAPE-42
- Severity of PTSD symptoms: PCL-5
- Severity of depression and anxiety: PHQ-9 and GAD-7
- Impaired functioning: WSAS
- Health status: EQ-5D-L
- Drug use: DAST 10
- Medication use
- Resource data use - we will ask questions about resource use including information on the use of primary and secondary care services, use of social services and disability payments received, time off school or work, and productivity loss due to time off work or study.

If there are difficulties following up trial participants to obtain outcome data, researchers will prioritise collection of data on psychotic symptoms, PTSD symptoms and medication. Following the switch to single arm design, it will no longer be possible to blind researchers to treatment allocation in terms of avoiding potential bias in outcome measurement.

Quantitative data analysis

Quantitative data will be analysed in Stata. If there are no differences in baseline characteristics of those who were randomised and those who enter the single arm study, we will combine data to generate summary statistics on: (1) the proportion consenting to take part in the study; (2) the proportion completing the baseline assessment and agreeing to take part in the study; (3) the number of EMDR sessions attended and the proportion completing 8 or more sessions (regarded as an adequate dose of therapy); (4) the proportion completing the

For peer review only - http://bmjopen.bmj.com/site/about/guidelines.xhtml

follow-up assessments. Feasibility outcomes such as recruitment and retention rates will be calculated with 95% confidence intervals using the exact binomial method.

We will also report the proportion of individuals who transitioned to psychosis at 12 months, and the confidence intervals for the effect size will be calculated to assist the planning of a future RCT. We will also compare the continuous outcomes such as the severity of psychotic symptoms, depression, anxiety, PTSD symptoms and quality of life to show the frequency of data completion, mean and standard deviation for the three time points.

Qualitative data collection

We will conduct in-depth individual interviews with all the therapists and participants who participate in the trial. In addition, up to 10 individuals who declined to take part in the trial will be interviewed to explore their views and understanding of the study and EMDR, and their reasons for declining to take part.

To encourage participation, and because well-planned interviews can gather the same material as those conducted face-to-face,[35] therapists and study participants will be given the choice of being interviewed in person or over the telephone. Therapists will be interviewed within a month of finishing intervention delivery. The interviews will explore therapist views and experiences of delivering EMDR to patients with ARMS, how treatment could be better tailored to this patient group, and what resources they would need if the intervention was evaluated in a large scale RCT. The interviews will also be used to explore their views of the training and supervision received.

Interviews with participants who completed treatment or stopped early will explore their views and experiences of the treatment received, identify how treatment delivery could be refined to increase its acceptability, and how study materials could be improved. Participants who completed the study will be interviewed within a month of completing their 4-month follow-up, and those who stopped early will be interviewed within a month of doing so.

Topic guides will be developed for each set of interviews to ensure consistency across data collection. The therapist and study participant guides will be developed in parallel to ensure key areas are discussed with both practitioners and patients. This will help compare findings across the interviews, highlighting similarities and differences between the views and experiences of patients and practitioners, and increasing the confidence with which study conclusions can be drawn. With interviewee consent, the interviews will be audio-recorded and transcribed verbatim.

For peer review only - http://bmjopen.bmj.com/site/about/guidelines.xhtml

Qualitative data analysis

All interviews will be fully transcribed and anonymised. Data collection and analysis will proceed in parallel, so that insights from earlier data can shape later data collection, and enable the team to establish when data saturation has been reached. The data gathered will be analysed thematically. Transcripts will be independently coded by different researchers, who will then meet to discuss their coding and interpretation of the data. This will help to control for researcher bias and may lead to the coding frame being revised or defined more clearly. Transcripts will then be imported into the software package NVivo to allow electronic coding and retrieval of data. Once all the transcripts have been coded, data will be analysed using an approach similar to framework analysis.[36]

Additional qualitative work to address issues with recruitment and explore treatment options in ARMS populations

As the study progressed, to explore why recruitment had been so low, additional objectives were incorporated. These were:

1) To explore how potential ARMS patients are identified and managed in primary and secondary care services
2) To identify referral routes between primary and secondary care
3) To explore other researchers' experiences of recruiting ARMS patients to research studies in the UK

This additional qualitative work will entail:

a. Interviews with GPs, clinicians from Primary Care Liaison Services (PCLS) and Early Intervention services
b. Interviews with patients who did not participate in the interventional part of the study but who have been identified as ARMS by the EI teams we are recruiting from
c. Interviews with researchers who have been involved in recruiting ARMS patients to research studies in the UK.

Summary of changes to the original feasibility study

There have been a number of changes to the original feasibility study. These are summarized below:

a) Change of study design from randomized trial to single arm trial
b) Decrease in sample size from 40 to 20 participants

For peer review only - http://bmjopen.bmj.com/site/about/guidelines.xhtml

c) Change in the measure used to assess the severity of psychotic symptoms at baseline and follow-up assessments from the Positive Scale of the PANSS to the CAARMS, as the latter is more sensitive

d) Extending the time between patients being identified as ARMS to the time when they can be recruited into the study from 3 to 12 months

e) Additional interviews with GPs, other clinicians from primary and secondary care services, ARMS patients and researchers to better understand identification and management of ARMS patients, including routes into primary and secondary services.

Study management

Data management

Completed questionnaires will be stored securely in compliance with University of Bristol Data Security policies and the General Data Protection Regulation (GDPR). Personal details will be entered onto a secure database held on the University of Bristol server, and non-identifiable data will be entered onto a secure web-based database. Data collected on paper will be identifiable only by Patient Identification Number (PIN). Information capable of identifying individuals will be held on the database with passwords restricted to authorised study staff only.

Data will be stored in the University of Bristol research data repository, and will be available upon reasonable request.

Patient and public involvement

Public and patient involvement (PPI) played an important role in this study. This research idea was initially presented at a PPI event which brought together people with lived experiences of psychosis, their carers, clinicians, academics and commissioners. Further on, this idea was presented at the Hearing Voices Network, and through these contacts we established a PPI group who gave us important feedback on the research question, the intervention, and which measures to use. We anticipate that the group, which is still active, will: (i) ensure that the views of patients who have had a psychotic illness are heard at every stage of the study; (ii) inform any developments of the intervention to make sure these are acceptable to the target population, (iii) ensure patient documentation is easy to understand to maximise response and retention rates; and (iv) help interpret and disseminate study findings.

For peer review only

MONITORING AND ADVERSE EVENTS

Adverse events

We use the recommendations for defining and reporting adverse events and harm as outlined by Parry, Crawford, & Duggan (2016). All serious adverse events will be reported to the Chief Investigator and the Trial Steering Committee (TSC). All SAEs of a related and unexpected nature will be reported to the main REC, in accordance with any procedures of the Sponsor. The decision to audit the trial rests with University Hospitals Bristol.

Trial oversight

A trial steering committee (TSC) will have independent oversight of the study, meeting at six monthly intervals during the course of the trial. The TSC will also take on data monitoring role for this feasibility study.

Dissemination policy

The results of this study will be disseminated through publications and conferences. Findings from this feasibility trial will be published in psychiatry journals compliant with NIHR Open Access policy, and presented to clinical staff, service users, and relevant Clinical Commissioning Groups.

DISCUSSION

To our knowledge, this is the first study to investigate the feasibility of conducting a large RCT to establish whether EMDR could prevent the onset of psychosis in people with an at-risk mental state. As psychotic illnesses usually occur in early adulthood and disrupt a key period of professional and social development, preventing psychosis would improve patients' outcomes, and also decrease the substantial economic burden of this condition.

The results of this feasibility trial will provide us with a better understanding of this patient group, and will provide us with information that will be used to inform whether progression to a full trial would be feasible. The nested qualitative study will address issues of patient and therapist views of the intervention, and provide new insights into treatment pathways and experiences of this patient group. If shown to be feasible, a full scale RCT will be conducted to examine the clinical and cost-effectiveness of EMDR to prevent the onset of psychosis in ARMS.

For peer review only - http://bmjopen.bmj.com/site/about/guidelines.xhtml

Feasibility trial status

Recruitment to this study opened in May 2018, and will close in April 2020. At the time of writing (February 2020), 12 participants have been recruited to the study. We expect to finish follow-up data collection by April 2021.

This article describes protocol version 4, dated 2.08.2019. When all regulatory approvals had been received, the protocol was version 1, dated 11.01.2018. Since then, two major amendments have been made with changes to incorporate additional qualitative work, decrease the sample size, and change the study design from a RCT to a single arm trial. The latter change took place in July 2019 prior to which we had recruited 6 participants.

Acknowledgements

We would like to thank Professor Jonathan Bisson, Professor Peter Jones and Dr Fiona Warren for overseeing the trial as part of our TSC group. The authors would also like to thank members of our PPI group Mr Bradley Jones, Ms Claire Barnard and Joanna Gigon for their feedback on the research question, intervention and measures to use, comments on the supporting documentation, and suggestions on how to improve recruitment and increase retention to the study.

Contributors

SZ, NW, KT and DS designed the study. SZ is the grant holder and Chief Investigator. SZ, NW and KT will oversee the scientific rigour of the study. NW will provide expertise in the management and analysis of the study. KT will oversee the nested qualitative study. DS will coordinate, implement and run the trial. CD will assist with running the trial. All authors contributed to refinement of the study protocol and approved the final manuscript.

Funding

This study was funded by the NIHR Biomedical Research Centre at University Hospitals Bristol NHS Foundation Trust and the University of Bristol. The views expressed in this publication are those of the author(s) and not necessarily those of the NHS, the National Institute for Health Research or the Department of Health. The sponsor of the study is the University of Bristol. The sponsor and study funders had no authority over the running of the trial, and all decisions rest with the trial team.

Competing interests

The authors declare no conflict of interests

For peer review only - http://bmjopen.bmj.com/site/about/guidelines.xhtml

Ethics approval

South West-Cornwall & Plymouth Research Ethics Committee (formerly South West-Exeter Research Ethics Committee), Reference 18/SW/0037, 29.03.2018.

Legend

Figure 1. Study flow chart for the feasibility trial (randomised and non-randomised components)

REFERENCES

1 Vos T, Abajobir AA, Abbafati C, *et al.* Global, regional, and national incidence, prevalence, and years lived with disability for 328 diseases and injuries for 195 countries, 1990-2016: A systematic analysis for the Global Burden of Disease Study 2016. *Lancet* 2017;**390**:1211–59. doi:10.1016/S0140-6736(17)32154-2

2 Yung AR, Yuen HP, McGorry PD, *et al.* Mapping the onset of psychosis: the Comprehensive Assessment of At-Risk Mental States. *Aust N Z J Psychiatry* 2005;**39**:964–71. doi:10.1080/j.1440-1614.2005.01714.x

3 Fusar-Poli P. Predicting Psychosis. *Arch Gen Psychiatry* 2012;**69**:220. doi:10.1001/archgenpsychiatry.2011.1472

4 National Institute for Health and Care Excellence [NICE]. Psychosis and schizophrenia in adults: prevention and management. Retrieved from https://www.nice.org.uk/guidance/cg178/chapter/1-recommendations#preventing-psychosis-2. Retrieved from https//www.nice.org.uk/guidance/cg178/chapter/1-recommendations#preventing-psychosis-2 (accessed Jan 2020). 2014.

5 Stafford MR, Jackson H, Mayo-Wilson E, *et al.* Early interventions to prevent psychosis: Systematic review and meta-analysis. *BMJ* 2013;**346**:1–13. doi:10.1136/bmj.f185

6 Varese F, Smeets F, Drukker M, *et al.* Childhood adversities increase the risk of psychosis: A meta-analysis of patient-control, prospective-and cross-sectional cohort studies. *Schizophr Bull* 2012;**38**:661–71. doi:10.1093/schbul/sbs050

7 Kraan T, Velthorst E, Smit F, *et al.* Trauma and recent life events in individuals at ultra high risk for psychosis: Review and meta-analysis. *Schizophr Res* 2015;**161**:143–9. doi:10.1016/j.schres.2014.11.026

8 Matheson SL, Shepherd AM, Pinchbeck RM, *et al.* Childhood adversity in schizophrenia: A systematic meta-analysis. *Psychol Med* 2013;**43**:225–38.

For peer review only - http://bmjopen.bmj.com/site/about/guidelines.xhtml

doi:10.1017/S0033291712000785

9 Zammit S, Lewis C, Dawson S, *et al.* Undetected post-traumatic stress disorder in secondary-care mental health services: Systematic review. *Br J Psychiatry* 2018;**212**:11–8. doi:10.1192/bjp.2017.8

10 Trotta A, Murray RM, Fisher HL. The impact of childhood adversity on the persistence of psychotic symptoms: a systematic review and meta-analysis. *Psychol Med* 2015;**45**:2481–98. doi:10.1017/S0033291715000574

11 Van Dam DS, Van Der Ven E, Velthorst E, *et al.* Childhood bullying and the association with psychosis in non-clinical and clinical samples: A review and meta-analysis. *Psychol Med* 2012;**42**:2463–76. doi:10.1017/S0033291712000360

12 Croft J, Heron J, Teufel C, *et al.* Association of Trauma Type, Age of Exposure, and Frequency in Childhood and Adolescence with Psychotic Experiences in Early Adulthood. *JAMA Psychiatry* 2019;**76**:79–86. doi:10.1001/jamapsychiatry.2018.3155

13 Chen L, Zhang G, Hu M, *et al.* Eye movement desensitization and reprocessing versus cognitive-behavioral therapy for adult posttraumatic stress disorder: Systematic review and meta-analysis. *J Nerv Ment Dis* 2015;**203**:443–51. doi:10.1097/NMD.0000000000000306

14 Bisson J, Andrew M. Psychological treatment of post-traumatic stress disorder (PTSD). *Cochrane database Syst Rev* 2007;:CD003388. doi:10.1002/14651858.CD003388.pub3

15 Seidler GH, Wagner FE. Comparing the efficacy of EMDR and trauma-focused cognitive-behavioral therapy in the treatment of PTSD: A meta-analytic study. *Psychol Med* 2006;**36**:1515–22. doi:10.1017/S0033291706007963

16 Watts B V., Schnurr PP, Mayo L, *et al.* Meta-analysis of the efficacy of treatments for posttraumatic stress disorder. *J Clin Psychiatry* 2013;**74**:541–50. doi:10.4088/JCP.12r08225

17 Barnes M, Wiles N, Morrison J, *et al.* Exploring patients’ reasons for declining contact in a cognitive behavioural therapy randomised controlled trial in primary care. *Br J Gen Pract* 2012;**62**:371–7. doi:10.3399/bjgp12X641492

18 Addington J, Epstein I, Liu L, *et al.* A randomized controlled trial of cognitive behavioral therapy for individuals at clinical high risk of psychosis. *Schizophr Res* 2011;**125**:54–61. doi:10.1016/j.schres.2010.10.015

19 RCPsych. Report of the Second Round of the National Audit of Schizophrenia (NAST). Retrieved from

For peer review only - http://bmjopen.bmj.com/site/about/guidelines.xhtml

http://www.rcpsych.ac.uk/workinpsychiatry/qualityimprovement/nationalclinicalaudits/nationalschizophreniaaudit/reports.aspx. 2014.

20 De Bont PAJM, Van Den Berg DPG, Van Der Vleugel BM, *et al.* Prolonged exposure and EMDR for PTSD v. a PTSD waiting-list condition: Effects on symptoms of psychosis, depression and social functioning in patients with chronic psychotic disorders. *Psychol Med* 2016;**46**:2411–21. doi:10.1017/S0033291716001094

21 Bovin MJ, Marx BP, Weathers FW, *et al.* Psychometric properties of the PTSD Checklist for Diagnostic and Statistical Manual of Mental Disorders-Fifth Edition (PCL-5) in veterans. *Psychol Assess* 2016;**28**:1379–91. doi:10.1037/pas0000254

22 Gray MJ, Litz BT, Hsu JL, *et al.* Psychometric properties of the life events checklist. *Assessment* 2004;**11**:330–41. doi:10.1177/1073191104269954

23 Bernstein DP, Fink L. *Childhood trauma questionnaire : a retrospective self-report : manual*. Orlando: : Psychological Corporation 1998.

24 Kay SR, Fiszbein A, Opler LA. The positive and negative syndrome scale (PANSS) for schizophrenia. *Schizophr Bull* 1987;**13**:261–76. doi:10.1093/schbul/13.2.261

25 Haddock G, McCarron J, Tarrier N, *et al.* Scales to measure dimensions of hallucinations and delusions: the psychotic symptom rating scales (PSYRATS). *Psychol Med* 1999;**29**:879–89. doi:10.1017/s0033291799008661

26 Stefanis NC, Hanssen M, Smirnis NK, *et al.* Evidence that three dimensions of psychosis have a distribution in the general population. *Psychol Med* 2002;**32**:347–58. doi:10.1017/s0033291701005141

27 Kroenke K, Spitzer RL, Williams JB. The PHQ-9: validity of a brief depression severity measure. *J Gen Intern Med* 2001;**16**:606–13. doi:10.1046/j.1525-1497.2001.016009606.x

28 Spitzer RL, Kroenke K, Williams JBW, *et al.* A brief measure for assessing generalized anxiety disorder: the GAD-7. *Arch Intern Med* 2006;**166**:1092–7. doi:10.1001/archinte.166.10.1092

29 Mundt JC, Marks IM, Shear MK, *et al.* The Work and Social Adjustment Scale: a simple measure of impairment in functioning. *Br J Psychiatry* 2002;**180**:461–4. doi:10.1192/bjp.180.5.461

30 Herdman M, Gudex C, Lloyd A, *et al.* Development and preliminary testing of the new five-level version of EQ-5D (EQ-5D-5L). *Qual Life Res* 2011;**20**:1727–36. doi:10.1007/s11136-011-9903-x

31 Skinner HA. The drug abuse screening test. *Addict Behav* 1982;**7**:363–71.

For peer review only - http://bmjopen.bmj.com/site/about/guidelines.xhtml

doi:10.1016/0306-4603(82)90005-3

32 Ward-Brown J, Keane D, Bhutani G, *et al.* TF-CBT and EMDR for young people with trauma and first episode psychosis (using a phasic treatment approach): two early intervention service case studies. *Cogn Behav Ther* 2018;**11**:e17. doi:DOI: 10.1017/S1754470X18000193

33 Leeds A. *A Guide to the Standard EMDR Protocols for Clinicians, Supervisors, and Consultants*. 2nd ed. New York: : Springer Publishing Company 2016.

34 Teare MD, Dimairo M, Shephard N, *et al.* Sample size requirements to estimate key design parameters from external pilot randomised controlled trials: a simulation study. *Trials* 2014;**15**:264. doi:10.1186/1745-6215-15-264

35 Sturges JE, Hanrahan KJ. Comparing Telephone and Face-to-Face Qualitative Interviewing: a Research Note. *Qual Res* 2004;**4**:107–18. doi:10.1177/1468794104041110

36 Ritchie J, Spencer L. Analyzing Qualitative Data. In: Bryman A, Burgess R, eds. *Analyzing Qualitative Data*. London: 1994. 173–94.

37 Parry GD, Crawford MJ, Duggan C. Iatrogenic harm from psychological therapies- time to move on. *Br J Psychiatry* 2016;**208**:210–2. doi:10.1192/bjp.bp.115.163618

For peer review only

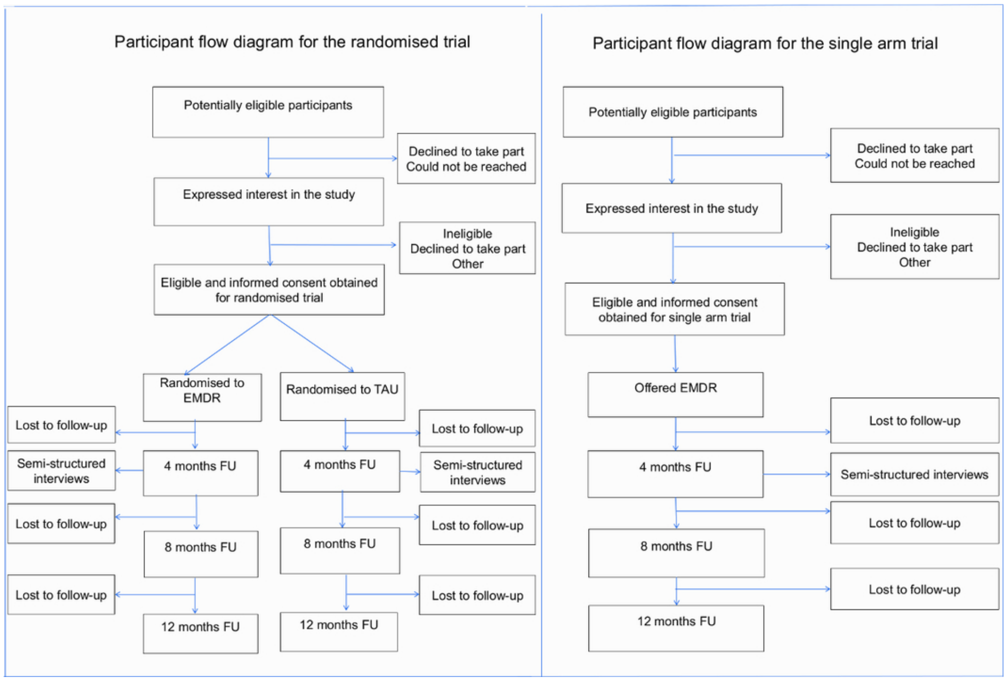

Figure 1. Study flow chart for the feasibility trial (randomised and non-randomised components)

89x60mm (300 x 300 DPI)

SPIRIT

STANDARD PROTOCOL ITEMS: RECOMMENDATIONS FOR INTERVENTIONAL TRIALS

SPIRIT 2013 Checklist: Recommended items to address in a clinical trial protocol and related documents*

Section/item	Item No	Description
**Administrative information**		
Title	1	Descriptive title identifying the study design, population, interventions, and, if applicable, trial acronym<br>- Page 1
Trial registration	2a	Trial identifier and registry name. If not yet registered, name of intended registry<br>- Page 2
	2b	All items from the World Health Organization Trial Registration Data Set<br>- Please see: https://www.isrctn.com/ISRCTN31976295?q=&filters=&sort=&offset=3&totalResults=16965&page=1&pageSize=10&searchType=basic-search
Protocol version	3	Date and version identifier<br>- Page 14
Funding	4	Sources and types of financial, material, and other support<br>- Page 14
Roles and responsibilities	5a	Names, affiliations, and roles of protocol contributors<br>- Page 1, 14
	5b	Name and contact information for the trial sponsor<br>- Page 14
	5c	Role of study sponsor and funders, if any, in study design; collection, management, analysis, and interpretation of data; writing of the report; and the decision to submit the report for publication, including whether they will have ultimate authority over any of these activities<br>- Page 14

For peer review only

	5d	Composition, roles, and responsibilities of the coordinating centre, steering committee, endpoint adjudication committee, data management team, and other individuals or groups overseeing the trial, if applicable (see Item 21a for data monitoring committee)<br>- Our Trial Steering Committee (TSC) has got three external members (trialist, psychosis expert and statistician). The role of the TSC is to provide oversight of the trial on behalf of the Trial Sponsor and funder. The main tasks of the TSC are: to provide expert advice on all aspects of the trial, to focus on the progress of the trial, adherence to the protocol, patient safety, to agree proposals for substantial protocol amendments, to monitor and review issues related to patient safety. The TSC will also take on data monitoring role for this feasibility study.
**Introduction**		
Background and rationale	6a	Description of research question and justification for undertaking the trial, including summary of relevant studies (published and unpublished) examining benefits and harms for each intervention<br>- Page 3-4
	6b	Explanation for choice of comparators<br>- Page 4, 7-8
Objectives	7	Specific objectives or hypotheses<br>- Page 4
Trial design	8	Description of trial design including type of trial (eg, parallel group, crossover, factorial, single group), allocation ratio, and framework (eg, superiority, equivalence, noninferiority, exploratory)<br>- Page 4-5
**Methods: Participants, interventions, and outcomes**		
Study setting	9	Description of study settings (eg, community clinic, academic hospital) and list of countries where data will be collected. Reference to where list of study sites can be obtained<br>- Page 5-6
Eligibility criteria	10	Inclusion and exclusion criteria for participants. If applicable, eligibility criteria for study centres and individuals who will perform the interventions (eg, surgeons, psychotherapists)<br>- Page 5
Interventions	11a	Interventions for each group with sufficient detail to allow replication, including how and when they will be administered<br>- Page 7-8

For peer review only

	11b	Criteria for discontinuing or modifying allocated interventions for a given trial participant (eg, drug dose change in response to harms, participant request, or improving/worsening disease)<br>- If a serious adverse event (SAE) occurs in relation to therapy, or participant has difficulties engaging with therapy to the point where therapy is no longer worthwhile, the therapy will be discontinued.
	11c	Strategies to improve adherence to intervention protocols, and any procedures for monitoring adherence (eg, drug tablet return, laboratory tests)<br>- For monitoring adherence, therapists in the study will inform members of the research team if participants have not attended therapy sessions.
	11d	Relevant concomitant care and interventions that are permitted or prohibited during the trial<br>- As mentioned in the exclusion criteria, participants receiving EMDR should not be currently receiving any other psychological therapy. Apart from this, participants in the trial can have access to any form of support/care that the NHS offers to patients at risk of psychosis.
Outcomes	12	Primary, secondary, and other outcomes, including the specific measurement variable (eg, systolic blood pressure), analysis metric (eg, change from baseline, final value, time to event), method of aggregation (eg, median, proportion), and time point for each outcome. Explanation of the clinical relevance of chosen efficacy and harm outcomes is strongly recommended<br>- Page 9
Participant timeline	13	Time schedule of enrolment, interventions (including any run-ins and washouts), assessments, and visits for participants. A schematic diagram is highly recommended (see Figure)<br>- See Figure 1.
Sample size	14	Estimated number of participants needed to achieve study objectives and how it was determined, including clinical and statistical assumptions supporting any sample size calculations<br>- Page 8-10
Recruitment	15	Strategies for achieving adequate participant enrolment to reach target sample size<br>- Page 5-6

Methods: Assignment of interventions (for controlled trials)

Allocation:

Sequence generation	16a	Method of generating the allocation sequence (eg, computer-generated random numbers), and list of any factors for stratification. To reduce predictability of a random sequence, details of any planned restriction (eg, blocking) should be provided in a separate document that is unavailable to those who enrol participants or assign interventions<br>- Page 8
Allocation concealment mechanism	16b	Mechanism of implementing the allocation sequence (eg, central telephone; sequentially numbered, opaque, sealed envelopes), describing any steps to conceal the sequence until interventions are assigned<br>- Page 8
Implementation	16c	Who will generate the allocation sequence, who will enrol participants, and who will assign participants to interventions<br>- Page 8
Blinding (masking)	17a	Who will be blinded after assignment to interventions (eg, trial participants, care providers, outcome assessors, data analysts), and how<br>- Page 8
	17b	If blinded, circumstances under which unblinding is permissible, and procedure for revealing a participant's allocated intervention during the trial<br>- Not applicable

Methods: Data collection, management, and analysis

Data collection methods	18a	Plans for assessment and collection of outcome, baseline, and other trial data, including any related processes to promote data quality (eg, duplicate measurements, training of assessors) and a description of study instruments (eg, questionnaires, laboratory tests) along with their reliability and validity, if known. Reference to where data collection forms can be found, if not in the protocol<br>- Please see page 6-7 for key references to questionnaires
	18b	Plans to promote participant retention and complete follow-up, including list of any outcome data to be collected for participants who discontinue or deviate from intervention protocols<br>- If there are any concerns about complete data collection, research team will prioritise collection of data on psychotic symptoms, PTSD symptoms and medication (using the instruments described on page 9.

Data management	19	Plans for data entry, coding, security, and storage, including any related processes to promote data quality (eg, double data entry; range checks for data values). Reference to where details of data management procedures can be found, if not in the protocol<br>- Page 12
Statistical methods	20a	Statistical methods for analysing primary and secondary outcomes. Reference to where other details of the statistical analysis plan can be found, if not in the protocol<br>- Page 9-11
	20b	Methods for any additional analyses (eg, subgroup and adjusted analyses)<br>- Page 11
	20c	Definition of analysis population relating to protocol non-adherence (eg, as randomised analysis), and any statistical methods to handle missing data (eg, multiple imputation)<br>- Data from the randomised and single arm trial will be analysed as described on page 9-10
**Methods: Monitoring**		
Data monitoring	21a	Composition of data monitoring committee (DMC); summary of its role and reporting structure; statement of whether it is independent from the sponsor and competing interests; and reference to where further details about its charter can be found, if not in the protocol. Alternatively, an explanation of why a DMC is not needed<br>- As this is a small feasibility trial, the responsibilities of the DMC will be taken over by the TSC; composition, roles and responsibilities of the TSC can be seen at Item 5d.
	21b	Description of any interim analyses and stopping guidelines, including who will have access to these interim results and make the final decision to terminate the trial<br>- N/A.
Harms	22	Plans for collecting, assessing, reporting, and managing solicited and spontaneously reported adverse events and other unintended effects of trial interventions or trial conduct<br>- Page 13
Auditing	23	Frequency and procedures for auditing trial conduct, if any, and whether the process will be independent from investigators and the sponsor<br>- The decision to audit the trial rests with University Hospitals Bristol (UHB) which is independent from investigators and sponsor.

For peer review only - http://bmjopen.bmj.com/site/about/guidelines.xhtml

Ethics and dissemination

Research ethics approval	24	Plans for seeking research ethics committee/institutional review board (REC/IRB) approval<br>- Ethics approval received as described on page 15
Protocol amendments	25	Plans for communicating important protocol modifications (eg, changes to eligibility criteria, outcomes, analyses) to relevant parties (eg, investigators, REC/IRBs, trial participants, trial registries, journals, regulators)<br>- Amendments to the original protocol have been discussed with the TSC. All relevant parties, including the ISRCTN, have been notified of changes to the protocol.
Consent or assent	26a	Who will obtain informed consent or assent from potential trial participants or authorised surrogates, and how (see Item 32)<br>- Written informed consent will be obtained by researchers in the trial, as described on page 6-7.
	26b	Additional consent provisions for collection and use of participant data and biological specimens in ancillary studies, if applicable<br>- Not applicable
Confidentiality	27	How personal information about potential and enrolled participants will be collected, shared, and maintained in order to protect confidentiality before, during, and after the trial<br>- Page 12
Declaration of interests	28	Financial and other competing interests for principal investigators for the overall trial and each study site<br>- The authors declare no conflict of interests
Access to data	29	Statement of who will have access to the final trial dataset, and disclosure of contractual agreements that limit such access for investigators<br>- Only the immediate research team will have access to the final trial dataset.
Ancillary and post-trial care	30	Provisions, if any, for ancillary and post-trial care, and for compensation to those who suffer harm from trial participation<br>- There will be no post-trial care for participants who were randomised to treatment as usual as part of the original design of the study. This has been communicated to participants upon enrolling in the trial.
Dissemination policy	31a	Plans for investigators and sponsor to communicate trial results to participants, healthcare professionals, the public, and other relevant groups (eg, via publication, reporting in results databases, or other data sharing arrangements), including any publication restrictions<br>- Page 13

For peer review

	31b	Authorship eligibility guidelines and any intended use of professional writers<br>- Authorship will follow the ICMJE recommendations (substantial contribution to the design of the study, analysis or data interpretation, drafting or revising the work, final approval or the published version and agreement to be accountable for all aspects of the work).
	31c	Plans, if any, for granting public access to the full protocol, participant-level dataset, and statistical code<br>- The full protocol can be accessed online at https://www.isrctn.com/ISRCTN31976295?q=&filters=&sort=&offset=3&totalResults=16965&page=1&pageSize=10&searchType=basic-search<br>- Participants gave consent for their data to be anonymised and made available for use in future studies. Data will be available upon reasonable request via the first author.
**Appendices**		
Informed consent materials	32	Model consent form and other related documentation given to participants and authorised surrogates<br>- Please see the attached Participant Information Sheet
Biological specimens	33	Plans for collection, laboratory evaluation, and storage of biological specimens for genetic or molecular analysis in the current trial and for future use in ancillary studies, if applicable<br>- Not applicable

*It is strongly recommended that this checklist be read in conjunction with the SPIRIT 2013 Explanation & Elaboration for important clarification on the items. Amendments to the protocol should be tracked and dated. The SPIRIT checklist is copyrighted by the SPIRIT Group under the Creative Commons "Attribution-NonCommercial-NoDerivs 3.0 Unported" license.

For peer review only

BMJ Open

A feasibility study of eye-movement desensitization and reprocessing (EMDR) in people with an at-risk mental state (ARMS) for psychosis: study protocol

Journal:	*BMJ Open*
Manuscript ID	bmjopen-2020-038620.R1
Article Type:	Protocol
Date Submitted by the Author:	27-Jul-2020
Complete List of Authors:	Strelchuk, Daniela; University of Bristol, Centre for Academic Mental Health, Population Health Sciences, Bristol Medical School; University Hospitals Bristol NHS Foundation Trust, National Institute for Health Research Bristol Biomedical Research Centre<br>Wiles, Nicola; University of Bristol, Centre for Academic Mental Health, Population Health Sciences, Bristol Medical School; University Hospitals Bristol NHS Foundation Trust, National Institute for Health Research Bristol Biomedical Research Centre<br>Turner, Katrina; University of Bristol, Centre for Academic Mental Health, Population Health Sciences, Bristol Medical School; University Hospitals Bristol NHS Foundation Trust, National Institute for Health Research Bristol Biomedical Research Centre<br>Derrick, Catherine; University of Bristol, Centre for Academic Mental Health, Population Health Sciences, Bristol Medical School<br>Zammit, Stan; University of Bristol, Centre for Academic Mental Health, Population Health Sciences, Bristol Medical School; Cardiff University, MRC Centre for Neuropsychiatric Genetics and Genomics, Division of Psychological Medicine and Clinical Neuroscience
**Primary Subject Heading**:	Mental health
Secondary Subject Heading:	Qualitative research, Evidence based practice
Keywords:	Schizophrenia & psychotic disorders < PSYCHIATRY, QUALITATIVE RESEARCH, Clinical trials < THERAPEUTICS

SCHOLARONE™
Manuscripts

For peer review only - http://bmjopen.bmj.com/site/about/guidelines.xhtml

I, the Submitting Author has the right to grant and does grant on behalf of all authors of the Work (as defined in the below author licence), an exclusive licence and/or a non-exclusive licence for contributions from authors who are: i) UK Crown employees; ii) where BMJ has agreed a CC-BY licence shall apply, and/or iii) in accordance with the terms applicable for US Federal Government officers or employees acting as part of their official duties; on a worldwide, perpetual, irrevocable, royalty-free basis to BMJ Publishing Group Ltd ("BMJ") its licensees and where the relevant Journal is co-owned by BMJ to the co-owners of the Journal, to publish the Work in this journal and any other BMJ products and to exploit all rights, as set out in our licence.

The Submitting Author accepts and understands that any supply made under these terms is made by BMJ to the Submitting Author unless you are acting as an employee on behalf of your employer or a postgraduate student of an affiliated institution which is paying any applicable article publishing charge ("APC") for Open Access articles. Where the Submitting Author wishes to make the Work available on an Open Access basis (and intends to pay the relevant APC), the terms of reuse of such Open Access shall be governed by a Creative Commons licence – details of these licences and which Creative Commons licence will apply to this Work are set out in our licence referred to above.

Other than as permitted in any relevant BMJ Author's Self Archiving Policies, I confirm this Work has not been accepted for publication elsewhere, is not being considered for publication elsewhere and does not duplicate material already published. I confirm all authors consent to publication of this Work and authorise the granting of this licence.

For peer review only

A feasibility study of eye-movement desensitization and reprocessing (EMDR) in people with an at-risk mental state (ARMS) for psychosis: study protocol

Daniela Strelchuk, MSc, Centre for Academic Mental Health, Population Health Sciences, Bristol Medical School, University of Bristol, Bristol, UK and National Institute for Health Research Bristol Biomedical Research Centre, University Hospitals Bristol NHS Foundation Trust and University of Bristol.

Nicola Wiles, PhD, Centre for Academic Mental Health, Population Health Sciences, Bristol Medical School, University of Bristol, Bristol, UK, and National Institute for Health Research Bristol Biomedical Research Centre, University Hospitals Bristol NHS Foundation Trust and University of Bristol.

Katrina Turner, PhD, Centre for Academic Mental Health, Population Health Sciences, Bristol Medical School, University of Bristol, Bristol, UK, and National Institute for Health Research Bristol Biomedical Research Centre, University Hospitals Bristol NHS Foundation Trust and University of Bristol.

Catherine Derrick, Centre for Academic Mental Health, Population Health Sciences, Bristol Medical School, University of Bristol, Bristol, UK

Stan Zammit, PhD, Centre for Academic Mental Health, Population Health Sciences, Bristol Medical School, University of Bristol, Bristol, UK; MRC Centre for Neuropsychiatric Genetics and Genomics, Division of Psychological Medicine and Clinical Neuroscience, Cardiff University, Cardiff, UK and National Institute for Health Research Bristol Biomedical Research Centre, University Hospitals Bristol NHS Foundation Trust and University of Bristol.

Corresponding author:

Daniela Strelchuk

Oakfield House, Oakfield Grove, Bristol, BS8 2BN;

Email: daniela.strelchuk@bristol.ac.uk

Tel: 07575540193

Word count: 3,994

For peer review only - http://bmjopen.bmj.com/site/about/guidelines.xhtml

Abstract

Introduction: Trauma can play an important role in the development of psychosis, yet no studies have investigated whether a trauma-focused psychological therapy could prevent the onset of psychosis in people at high risk of developing this condition. This study aims to establish whether it would be feasible to conduct a multi-centre randomised controlled trial (RCT) to investigate the clinical and cost effectiveness of eye movement desensitization and reprocessing therapy (EMDR) to prevent the onset of psychosis in people with an at-risk mental state (ARMS).

Methods/analysis: This is a single arm trial with a nested qualitative study where all participants (target n=20) will be offered EMDR. Eligible participants are those who meet criteria for ARMS; have experienced a traumatic event before the onset of ARMS symptomatology; and have at least one symptom of post-traumatic stress disorder (PTSD). Participants will be followed-up at 4, 8 and 12 months after the baseline assessment. The primary outcome measure is transition to psychosis, and secondary outcome measures include severity of psychotic symptoms, PTSD, depression, anxiety, impaired functioning, health status, and resource use. The analysis will aim to establish the rates of recruitment and retention for a large-scale RCT. Interviews with therapists and patients will explore their views of the study and their experiences of delivering or receiving EMDR.

Ethics and dissemination

This protocol has been approved by the South West-Cornwall & Plymouth Research Ethics Committee (Reference 18/SW/0037). Findings will be disseminated through journal publications, conference presentations and meetings with service users, their families, mental health professionals and commissioners.

Trial registration: ISRCTN31976295

Strengths and limitations of the study

- This is the first study to investigate the feasibility of conducting a large multi-centre RCT of EMDR for the prevention of psychosis in people with ARMS
- The study includes qualitative work to explore therapists' and patients' views of the treatment offered/received
- Therapy sessions follow an EMDR protocol specifically designed for people with psychosis
- The decision to change the study design to a single arm trial may limit our ability to draw robust conclusions about recruiting to an RCT

For peer review only - http://bmjopen.bmj.com/site/about/guidelines.xhtml

For peer review only

INTRODUCTION

Psychotic illnesses are one of the leading causes of disability worldwide.[1] The onset of psychosis is often preceded by a prodromal phase, known as an at-risk mental state (ARMS) for psychosis, which is characterized by a series of non-specific or attenuated psychotic symptoms.[2] Approximately 22% of ARMS individuals will transition to psychosis within one year and 36% of them within 3 years.[3]

Various interventions have been used for the prevention of psychosis, but there is still limited evidence on the effectiveness of these approaches. Clinical guidelines recommend cognitive-behavioural therapy (CBT) as a first-line treatment.[4] However, whilst a meta-analysis of randomized controlled trials showed that CBT has a moderate effect on transition to psychosis at 12 months follow-up (RR 0.64, 95% CI 0.44, 0.93), the evidence was of low to moderate quality and the effect was not maintained over the longer-term (18 months: RR 0.55, 95% CI 0.25, 1.19).[5]

Exposure to trauma is 2-3 times more common in ARMS or psychosis than in controls,[6–8] and post-traumatic stress disorder (PTSD) is also common, although not usually detected.[9] Despite strong evidence showing that trauma is a key factor in the development of psychosis,[10–12] no studies have yet investigated whether a trauma-focused therapy could prevent the onset of psychosis in ARMS.

Eye movement desensitisation and reprocessing (EMDR) is a trauma-focused therapy that has comparable efficacy to trauma-focused CBT (TF-CBT) in treating PTSD.[13–16] However, the two forms of therapy are different. While TF-CBT seeks to directly identify and restructure the negative beliefs, EMDR does this indirectly, relying more on the integration of emotional, cognitive, sensory, physical, and contextual information to allow adaptive processing of traumatic memories. Unlike TF-CBT, EMDR does not require patients to give a detailed description of the trauma, which may be distressing. Also, unlike TF-CBT, EMDR does not involve homework, which may make it more suitable for people with a disorganized lifestyle, such as those with ARMS.

To date, there has been only one randomised controlled trial (RCT) comparing EMDR with treatment as usual in patients with co-morbid psychosis and PTSD. This study showed that EMDR improved PTSD, and also reduced paranoid delusions, but did not reduce auditory hallucinations at 6 months follow-up.[17] However, the EMDR protocol did not specifically target psychotic symptoms. Furthermore, most participants had chronic forms of psychosis

For peer review only - http://bmjopen.bmj.com/site/about/guidelines.xhtml

and hence psychotic symptoms might have been less amenable to treatment than more recent onset symptoms present in ARMS subjects.

Objectives

The overarching aim of this study is to establish whether it would be feasible to conduct a large multi-centre RCT to evaluate the clinical and cost-effectiveness of EMDR to prevent the onset of psychosis in people with an at-risk mental state. The study has the following objectives:

1. To estimate the rate of recruitment and retention to inform the large-scale RCT
2. To refine the eligibility criteria, screening and recruitment procedures
3. To optimize the EMDR protocols and learn about the factors which affect the implementation of EMDR
4. To explore patients' and therapists' views of EMDR as a treatment for ARMS
5. To investigate patients' and therapists' views of the study design and study materials
6. To understand what treatment as usual (TAU) consists of for patients with ARMS

METHODS AND ANALYSIS

Study design

The study consists of a single arm feasibility trial with a nested qualitative study, and is conducted in collaboration with the Early Intervention (EI) teams from the Avon and Wiltshire Mental Health Partnership NHS Trust (AWP). All participants will be offered EMDR, and will be assessed at baseline, 4, 8 and 12 months follow-up as described below.

The study design outlined is the final version of the trial protocol. The original protocol was changed as a result of low recruitment. Recruitment challenges are described later.

Inclusion and exclusion criteria

Inclusion criteria

- Aged 16 years or over who are at risk of psychosis (as defined in the Comprehensive Assessment of At-Risk Mental States (CAARMS))[2]
- Presence of at least one positive symptom of psychosis (perceptual abnormality, unusual thought content, non-bizarre ideas or disorganised speech) scored ≥3 on CAARMS
- History of traumatic experience as defined in ICD-10 F43.1, occurring prior to onset of first positive symptom

For peer review only - http://bmjopen.bmj.com/site/about/guidelines.xhtml

- Presence of one or more symptoms of re-living, avoidance, hyper-arousal, or cognitive distortions in relation to the traumatic experience (assessed using the PTSD Checklist for DSM-V (PCL5)) during the last month.[18]

Exclusion criteria

- History of treated or untreated psychotic illness or learning disability
- Currently taking antipsychotics
- Currently receiving psychological therapy
- Completed a trauma-focused psychological therapy in the last 2 years
- Insufficient fluency in English
- Lacking mental capacity to provide valid informed consent

Recruitment procedure

Participants will be identified via two routes:

1. Early Intervention services

Researchers will identify clinical teams who are willing to identify potential participants via the following routes:

a) during routine clinic appointments - clinicians will introduce the study to patients who have met ARMS criteria within the past 12 months. Interested patients will be asked to complete section 1 of an Expression of Interest (EOI) form, and to provide their contact details. Completed EOI forms will be returned to the research team by secure email. A member of the research team will telephone interested individuals and, if the individual is willing to take part, agree a time and place for the researcher to meet with them to complete the eligibility assessment. Following the telephone call, the researcher will post the individual a full participant information sheet and a letter confirming the appointment.

Individuals who do not want to hear more about the study will be asked to complete section 2 of the EOI form, which asks for information on age, gender, reasons for non-participation and willingness to take part in a short interview (see 'qualitative data collection' for more detail).

b) postal or telephone invitations from clinicians – If clinicians do not introduce the study to patients during the appointment, they will post ARMS patients a brief information sheet about the study, an EOI form and a stamped addressed envelope. Clinicians may also introduce the study to patients over the telephone. If the patient is interested, the clinician will ask the patient for permission to share their contact details with the research team. The clinician will then fill in the "Phone permission to contact" form and will email it to the research team. Recruitment

For peer review only - http://bmjopen.bmj.com/site/about/guidelines.xhtml

will then proceed as outlined in (a).

c) postal invitations sent out by NIHR CRN Clinical Studies Officers (CSO) - CSOs will pre-screen potential participants using the electronic patient record system (RIO), and liaise with the care team regarding potentially eligible patients who will then be sent a brief information sheet and details on how to contact the research team if they are interested in the study. Patients who do not reply will be followed up by telephone by the CSO within a week of receiving the documents. If the patient is interested in the study, the CSO will take permission over the phone to share their contact details with the research team. Once the research team have received contact details, recruitment will proceed as outlined in (a).

2. Everyone included within AWP

'Everyone Included' is a standard approach to research in AWP, whereby service users are routinely informed about relevant research opportunities. The AWP Research and Development department will send a 'Research Opportunity Letter' on behalf of the research team to potential participants briefly explaining the study and what it involves. A researcher will telephone individuals who are interested in the study and answer any questions. If the individual is willing to take part, an eligibility assessment will be arranged.

Consent and baseline eligibility assessment

Individuals interested in taking part will be invited to a face-to-face appointment with a researcher to establish eligibility, answer questions about participation, and obtain written informed consent.

The process of consenting young people (16 and 17 years old) will follow the Department of Health Reference Guide to Consent[19] and the Good Medical Practice Guidelines,[20] which provide the guide that all health professionals need to take into account in obtaining consent. Although young people may be more vulnerable than adults, they are presumed to be able to give consent to their treatment. As in the case of adults, if young people decline to take part in the study their decision will be fully respected and accepted.

In order to establish eligibility, patients will be asked to complete the following scales:

- Life-events Checklist for DSM-5 (LEC-5)[21]
- Childhood Trauma Questionnaire (CTQ)[22]
- PTSD Checklist for DSM-5 (PCL5)[18]

For peer review only - http://bmjopen.bmj.com/site/about/guidelines.xhtml

Those who are eligible will be asked to provide written informed consent for their participation in the trial and sociodemographic information. Additional baseline data will be collected using the following quantitative measures:

- Severity of psychotic symptoms: CAARMS,[2] the Negative Scale of the Positive and Negative Syndrome Scale (PANSS),[23] the Psychotic Symptom Rating Scales (PSYRAT)[24] and the Community Assessment of Psychic Experiences (CAPE-42).[25]
- Severity of depression and anxiety: Patient Health questionnaire (PHQ-9) for depression[26] and Generalized Anxiety Disorder Questionnaire (GAD-7) for anxiety[27]
- Impaired functioning: Work and Social Adjustment Scale (WSAS)[28]
- Health status: Five-level version of the EQ-5D (EQ-5D-5L)[29]
- Drug use: The Drug Abuse Screening Test (DAST 10)[30]

Data will also be collected on medication use via self-report measures. The baseline eligibility assessment will last about 90 minutes.

Intervention: EMDR

Participants will receive up to 12 sessions of manualized, weekly, face-to-face EMDR therapy.[31] Treatment has been manualized by EMDR consultants from Lancashire and South Cumbria Care NHS Foundation Trust (LSCFT) and individualised to target psychotic symptoms. The treatment protocol was developed and evaluated through pilot cases with first episode psychosis clients,[32] and further refined as part of an ongoing feasibility randomised controlled trial funded by the National Institute of Health Research (PB-PG-0317-20037; ISRCTN43816889). Therapeutic sessions will be delivered by EMDR UK & Europe trained therapists who will receive training based on the LSCFT manual for the study. The training will be tailored to therapists' needs, and will last between 4 to 8 hours. All EMDR therapists will have at least one year of experience of providing EMDR, and monthly supervision with an EMDR consultant will be in place. Each session will last approximately 90 minutes. The first 2-4 sessions will focus on establishing the therapeutic alliance, preparation for EMDR and stabilization techniques, assessment and identification of targets, cognitions, emotions and bodily sensations. The following 8-10 sessions will focus on desensitisation, installation of positive cognitions and body scan for past, present and future stressful situations.

Written permission will be sought to audio-record the therapeutic sessions. 10% of the sessions will be randomly sampled and evaluated by accredited EMDR therapists

For peer review only - http://bmjopen.bmj.com/site/about/guidelines.xhtml

independent of the study, to check treatment fidelity, using the EMDR fidelity checklist.[33] Therapists will be encouraged to liaise with participants' care coordinators as this could improve participants' adherence to therapy. If a serious adverse event (SAE) occurs in relation to therapy, or participant has difficulties engaging with therapy to the point where therapy is no longer worthwhile, the therapy will be discontinued.

Challenges to recruitment

The original feasibility study was designed as a RCT comparing EMDR with treatment as usual (TAU). TAU in AWP is quite heterogeneous and consists of discharge back to the referrer, signposting to other services, CBT, family intervention or psycho-education. Randomisation took place using a remote computerised randomisation service administered by the Bristol Trials Centre ensuring that allocations were concealed from the recruiting researcher. Randomisation was minimized by psychotic symptom severity (score on positive symptoms on CAARMS <11 or ≥11). However, following challenges to recruitment (described below), the study design was changed to a single arm study.

During the first seven months of the study (May to December 2018), we only recruited three participants (against a target of 21). This poor recruitment was primarily because the number of patients identified as ARMS by the EI teams within AWP was much lower than expected, and only three of the six EI teams we originally planned to recruit from were able to support the study due to financial constraints to managing ARMS clients. Therefore, from July 2019, all participants recruited to the study were offered EMDR. Figure 1 shows the flow of participants in the randomised and single arm phases of the trial.

Sample size and feasibility of recruitment

In the original protocol we had planned to recruit up to 40 participants. The initial sample size was informed by literature guidelines[34] and pragmatic considerations concerning potential difficulties accessing this population. The feasibility study would not be powered to detect important clinical differences between EMDR and TAU groups but would provide estimates of the completion and retention rates that would further assist in planning the recruitment for a future RCT.

Given the challenges with recruitment and modification of our study design (from a randomised to single arm trial), we decreased our target sample size from 40 to 20 participants. This sample size will provide us with reasonable precision around estimates of percentages completing the intervention and follow-ups. For example, if 70% of those who started EMDR complete the intervention, the 95% confidence intervals would be 45% and 88%. Similarly, if 75% of those who started EMDR are followed up to 12 months, the 95% confidence intervals

For peer review only - http://bmjopen.bmj.com/site/about/guidelines.xhtml

for the retention rate would be 50% and 91%.

Outcome assessment

The follow-up schedule will involve face-to-face assessments at 4, 8 and 12 months after the baseline assessment. Each follow-up assessment will last about 60 minutes. The primary outcome will be collected at 12 months follow-up, and the secondary outcome at 4, 8 and 12 months.

Primary outcome

- Transition to psychosis: ICD-10 diagnosis of psychotic disorder from clinical records or via the CAARMS

Secondary outcome

- Severity of psychotic symptoms: CAARMS, PSYRAT, the negative scale of the PANSS and CAPE-42
- Severity of PTSD symptoms: PCL-5
- Severity of depression and anxiety: PHQ-9 and GAD-7
- Impaired functioning: WSAS
- Health status: EQ-5D-L
- Drug use: DAST 10
- Medication use
- Resource data use - we will ask questions about resource use including information on the use of primary and secondary care services, use of social services and disability payments received, time off school or work, and productivity loss due to time off work or study.

If there are difficulties following up trial participants to obtain outcome data, researchers will prioritise collection of data on psychotic symptoms, PTSD symptoms and medication. Following the switch to single arm design, it will no longer be possible to blind researchers to treatment allocation in terms of avoiding potential bias in outcome measurement.

Quantitative data analysis

Quantitative data will be analysed in Stata version 15.[35] If there are no differences in baseline characteristics of those who were randomised and those who enter the single arm study, we will combine data to generate summary statistics on: (1) the proportion consenting to take part in the study; (2) the proportion completing the baseline assessment and agreeing

For peer review only - http://bmjopen.bmj.com/site/about/guidelines.xhtml

to take part in the study; (3) the number of EMDR sessions attended and the proportion completing 8 or more sessions (regarded as an adequate dose of therapy); (4) the proportion completing the follow-up assessments. Feasibility outcomes such as recruitment and retention rates will be calculated with 95% confidence intervals using the exact binomial method.

We will also report the proportion of individuals who transitioned to psychosis at 12 months, and the confidence intervals for the effect size will be calculated to assist the planning of a future RCT. We will also compare the continuous outcomes such as the severity of psychotic symptoms, depression, anxiety, PTSD symptoms and quality of life to show the frequency of data completion, mean and standard deviation for the three time points.

Qualitative data collection

We will conduct in-depth individual interviews with all the therapists and participants who participate in the trial. In addition, up to 10 individuals who declined to take part in the trial will be interviewed to explore their views and understanding of the study and EMDR, and their reasons for declining to take part.

To encourage participation, and because well-planned interviews can gather the same material as those conducted face-to-face,[36] therapists and study participants will be given the choice of being interviewed in person or over the telephone. Therapists will be interviewed within a month of finishing intervention delivery. The interviews will explore therapist views and experiences of delivering EMDR to patients with ARMS, how treatment could be better tailored to this patient group, and what resources they would need if the intervention was evaluated in a large scale RCT. The interviews will also be used to explore their views of the training and supervision received.

Interviews with participants who completed treatment or stopped early will explore their views and experiences of the treatment received, identify how treatment delivery could be refined to increase its acceptability, and how study materials could be improved. Participants who completed the study will be interviewed within a month of completing their 4-month follow-up, and those who stopped early will be interviewed within a month of doing so.

Topic guides will be developed for each set of interviews to ensure consistency across data collection. The therapist and study participant guides will be developed in parallel to ensure key areas are discussed with both practitioners and patients. This will help compare findings across the interviews, highlighting similarities and differences between the views and experiences of patients and practitioners, and increasing the confidence with which study

For peer review only - http://bmjopen.bmj.com/site/about/guidelines.xhtml

For peer review only

conclusions can be drawn. With interviewee consent, the interviews will be audio-recorded and transcribed verbatim.

Qualitative data analysis

All interviews will be fully transcribed and anonymised. Data collection and analysis will proceed in parallel, so that insights from earlier data can shape later data collection, and enable the team to establish when data saturation has been reached. The data gathered will be analysed thematically. Transcripts will be independently coded by different researchers, who will then meet to discuss their coding and interpretation of the data. This will help to control for researcher bias and may lead to the coding frame being revised or defined more clearly. Transcripts will then be imported into the software package NVivo version 12[37] to allow electronic coding and retrieval of data. Once all the transcripts have been coded, data will be analysed using an approach similar to framework analysis.[38]

Additional qualitative work to address issues with recruitment and explore treatment options in ARMS populations

As the study progressed, to explore why recruitment had been so low, additional objectives were incorporated. These were:

1) To explore how potential ARMS patients are identified and managed in primary and secondary care services
2) To identify referral routes between primary and secondary care
3) To explore other researchers' experiences of recruiting ARMS patients to research studies in the UK

This additional qualitative work will entail:

a. Interviews with GPs, clinicians from Primary Care Liaison Services (PCLS) and Early Intervention services
b. Interviews with patients who did not participate in the interventional part of the study but who have been identified as ARMS by the EI teams we are recruiting from
c. Interviews with researchers who have been involved in recruiting ARMS patients to research studies in the UK.

Summary of changes to the original feasibility study

There have been a number of changes to the original feasibility study. These are summarized below:

For peer review only - http://bmjopen.bmj.com/site/about/guidelines.xhtml

a) Change of study design from randomized trial to single arm trial
b) Decrease in sample size from 40 to 20 participants
c) Change in the measure used to assess the severity of psychotic symptoms at baseline and follow-up assessments from the Positive Scale of the PANSS to the CAARMS, as the latter is more sensitive
d) Extending the time between patients being identified as ARMS to the time when they can be recruited into the study from 3 to 12 months
e) Additional interviews with GPs, other clinicians from primary and secondary care services, ARMS patients and researchers to better understand identification and management of ARMS patients, including routes into primary and secondary services.

Study management

Data management

Completed questionnaires will be stored securely in compliance with University of Bristol Data Security policies and the General Data Protection Regulation (GDPR). Personal details will be entered onto a secure database held on the University of Bristol server, and non-identifiable data will be entered onto a secure web-based database. Data collected on paper will be identifiable only by Patient Identification Number (PIN). Information capable of identifying individuals will be held on the database with passwords restricted to authorised study staff only.

Data will be stored in the University of Bristol research data repository, and will be available upon reasonable request.

Patient and public involvement

Public and patient involvement (PPI) played an important role in this study. This research idea was initially presented at a PPI event which brought together people with lived experiences of psychosis, their carers, clinicians, academics and commissioners. Further on, this idea was presented at the Hearing Voices Network, and through these contacts we established a PPI group who gave us important feedback on the research question, the intervention, and which measures to use. We anticipate that the group, which is still active, will: (i) ensure that the views of patients who have had a psychotic illness are heard at every stage of the study; (ii) inform any developments of the intervention to make sure these are acceptable to the target population, (iii) ensure patient documentation is easy to understand to maximise response and retention rates; and (iv) help interpret and disseminate study findings.

For peer review only - http://bmjopen.bmj.com/site/about/guidelines.xhtml

MONITORING AND ADVERSE EVENTS

Adverse events

We use the recommendations for defining and reporting adverse events and harm as outlined by Parry, Crawford, & Duggan (2016).[39] All serious adverse events will be reported to the Chief Investigator and the Trial Steering Committee (TSC). All SAEs of a related and unexpected nature will be reported to the main REC, in accordance with any procedures of the Sponsor. The decision to audit the trial rests with University Hospitals Bristol.

Trial oversight

A trial steering committee (TSC) will have independent oversight of the study, meeting at six monthly intervals during the course of the trial. The TSC will also take on data monitoring role for this feasibility study.

Dissemination policy

The results of this study will be disseminated through publications and conferences. Findings from this feasibility trial will be published in psychiatry journals compliant with NIHR Open Access policy, and presented to clinical staff, service users, and relevant Clinical Commissioning Groups.

DISCUSSION

To our knowledge, this is the first study to investigate the feasibility of conducting a large RCT to establish whether EMDR could prevent the onset of psychosis in people with an at-risk mental state. As psychotic illnesses usually occur in early adulthood and disrupt a key period of professional and social development, preventing psychosis would improve patients' outcomes, and also decrease the substantial economic burden of this condition.

The results of this feasibility trial will provide us with a better understanding of this patient group, and will provide us with information that will be used to inform whether progression to a full trial would be feasible. The nested qualitative study will address issues of patient and therapist views of the intervention, and provide new insights into treatment pathways and experiences of this patient group. If shown to be feasible, a full scale RCT will be conducted to examine the clinical and cost-effectiveness of EMDR to prevent the onset of psychosis in ARMS.

For peer review only

Feasibility trial status

Recruitment to this study opened in May 2018, and closed in May 2020. At the time of writing (July 2020), 14 participants have been recruited to the study. We expect to finish follow-up data collection by May 2021.

This article describes protocol version 4, dated 2.08.2019. When all regulatory approvals had been received, the protocol was version 1, dated 11.01.2018. Since then, two major amendments have been made with changes to incorporate additional qualitative work, decrease the sample size, and change the study design from a RCT to a single arm trial. The latter change took place in July 2019 prior to which we had recruited 6 participants.

Acknowledgements

We would like to thank Professor Jonathan Bisson, Professor Peter Jones and Dr Fiona Warren for overseeing the trial as part of our TSC group. The authors would also like to thank members of our PPI group Mr Bradley Jones, Ms Claire Barnard and Joanna Gigon for their feedback on the research question, intervention and measures to use, comments on the supporting documentation, and suggestions on how to improve recruitment and increase retention to the study.

Contributors

SZ, NW, KT and DS designed the study. SZ is the grant holder and Chief Investigator. SZ, NW and KT will oversee the scientific rigour of the study. NW will provide expertise in the management and analysis of the study. KT will oversee the nested qualitative study. DS will coordinate, implement and run the trial. CD will assist with running the trial. All authors contributed to refinement of the study protocol and approved the final manuscript.

Funding

This study was funded by the NIHR Biomedical Research Centre at University Hospitals Bristol NHS Foundation Trust (BRC-1215-20011) and the University of Bristol. The views expressed in this publication are those of the author(s) and not necessarily those of the NHS, the National Institute for Health Research or the Department of Health. The sponsor of the study is the University of Bristol. The sponsor and study funders had no authority over the running of the trial, and all decisions rest with the trial team.

Competing interests

The authors declare no conflict of interests

For peer review only - http://bmjopen.bmj.com/site/about/guidelines.xhtml

For peer review only

Ethics approval

South West-Cornwall & Plymouth Research Ethics Committee (formerly South West-Exeter Research Ethics Committee), Reference 18/SW/0037, 29.03.2018.

Legend

Figure 1. Study flow chart for the feasibility trial (randomised and non-randomised components)

REFERENCES

1 Vos T, Abajobir AA, Abbafati C, *et al.* Global, regional, and national incidence, prevalence, and years lived with disability for 328 diseases and injuries for 195 countries, 1990-2016: A systematic analysis for the Global Burden of Disease Study 2016. *Lancet* 2017;**390**:1211–59. doi:10.1016/S0140-6736(17)32154-2

2 Yung AR, Yuen HP, McGorry PD, *et al.* Mapping the onset of psychosis: the Comprehensive Assessment of At-Risk Mental States. *Aust N Z J Psychiatry* 2005;**39**:964–71. doi:10.1111/j.1440-1614.2005.01714.x

3 Fusar-Poli P. Predicting Psychosis. *Arch Gen Psychiatry* 2012;**69**:220. doi:10.1001/archgenpsychiatry.2011.1472

4 National Institute for Health and Care Excellence [NICE]. Psychosis and schizophrenia in adults: prevention and management. Retrieved from https://www.nice.org.uk/guidance/cg178/chapter/1-recommendations#preventing-psychosis-2. Retrieved from https//www.nice.org.uk/guidance/cg178/chapter/1-recommendations#preventing-psychosis-2 (accessed Jan 2020). 2014.

5 Stafford MR, Jackson H, Mayo-Wilson E, *et al.* Early interventions to prevent psychosis: Systematic review and meta-analysis. *BMJ* 2013;**346**:1–13. doi:10.1136/bmj.f185

6 Varese F, Smeets F, Drukker M, *et al.* Childhood adversities increase the risk of psychosis: A meta-analysis of patient-control, prospective-and cross-sectional cohort studies. *Schizophr Bull* 2012;**38**:661–71. doi:10.1093/schbul/sbs050

7 Kraan T, Velthorst E, Smit F, *et al.* Trauma and recent life events in individuals at ultra high risk for psychosis: Review and meta-analysis. *Schizophr Res* 2015;**161**:143–9. doi:10.1016/j.schres.2014.11.026

8 Matheson SL, Shepherd AM, Pinchbeck RM, *et al.* Childhood adversity in schizophrenia: A systematic meta-analysis. *Psychol Med* 2013;**43**:225–38.

For peer review only - http://bmjopen.bmj.com/site/about/guidelines.xhtml

doi:10.1017/S0033291712000785
9 Zammit S, Lewis C, Dawson S, *et al.* Undetected post-traumatic stress disorder in secondary-care mental health services: Systematic review. *Br J Psychiatry* 2018;**212**:11–8. doi:10.1192/bjp.2017.8
10 Trotta A, Murray RM, Fisher HL. The impact of childhood adversity on the persistence of psychotic symptoms: a systematic review and meta-analysis. *Psychol Med* 2015;**45**:2481–98. doi:10.1017/S0033291715000574
11 Van Dam DS, Van Der Ven E, Velthorst E, *et al.* Childhood bullying and the association with psychosis in non-clinical and clinical samples: A review and meta-analysis. *Psychol Med* 2012;**42**:2463–76. doi:10.1017/S0033291712000360
12 Croft J, Heron J, Teufel C, *et al.* Association of Trauma Type, Age of Exposure, and Frequency in Childhood and Adolescence with Psychotic Experiences in Early Adulthood. *JAMA Psychiatry* 2019;**76**:79–86. doi:10.1001/jamapsychiatry.2018.3155
13 Chen L, Zhang G, Hu M, *et al.* Eye movement desensitization and reprocessing versus cognitive-behavioral therapy for adult posttraumatic stress disorder: Systematic review and meta-analysis. *J Nerv Ment Dis* 2015;**203**:443–51. doi:10.1097/NMD.0000000000000306
14 Bisson J, Andrew M. Psychological treatment of post-traumatic stress disorder (PTSD). *Cochrane database Syst Rev* 2007;:CD003388. doi:10.1002/14651858.CD003388.pub3
15 Seidler GH, Wagner FE. Comparing the efficacy of EMDR and trauma-focused cognitive-behavioral therapy in the treatment of PTSD: A meta-analytic study. *Psychol Med* 2006;**36**:1515–22. doi:10.1017/S0033291706007963
16 Watts B V., Schnurr PP, Mayo L, *et al.* Meta-analysis of the efficacy of treatments for posttraumatic stress disorder. *J Clin Psychiatry* 2013;**74**:541–50. doi:10.4088/JCP.12r08225
17 de Bont PAJMJM, van den Berg DPGG, Van Der Vleugel BM, *et al.* Prolonged exposure and EMDR for PTSD v. a PTSD waiting-list condition: effects on symptoms of psychosis, depression and social functioning in patients with chronic psychotic disorders. *Psychol Med* 2016;**46**:2411–21. doi:DOI: 10.1017/S0033291716001094
18 Bovin MJ, Marx BP, Weathers FW, *et al.* Psychometric properties of the PTSD Checklist for Diagnostic and Statistical Manual of Mental Disorders-Fifth Edition (PCL-5) in veterans. *Psychol Assess* 2016;**28**:1379–91. doi:10.1037/pas0000254
19 Department of Health and Social Care. Reference guide to consent for examination or

For peer review only - http://bmjopen.bmj.com/site/about/guidelines.xhtml

treatment (second edition). 2009.https://www.gov.uk/government/publications/reference-guide-to-consent-for-examination-or-treatment-second-edition (accessed 3 May 2020).

20 General Medical Council. https://www.gmc-uk.org/static/documents/content/Good_medical_practice_-_English_1215.pdf. 2013.

21 Gray MJ, Litz BT, Hsu JL, *et al.* Psychometric properties of the life events checklist. *Assessment* 2004;**11**:330–41. doi:10.1177/1073191104269954

22 Bernstein DP, Fink L. *Childhood trauma questionnaire : a retrospective self-report : manual*. Orlando: : Psychological Corporation 1998.

23 Kay SR, Fiszbein A, Opler LA. The positive and negative syndrome scale (PANSS) for schizophrenia. *Schizophr Bull* 1987;**13**:261–76. doi:10.1093/schbul/13.2.261

24 Haddock G, McCarron J, Tarrier N, *et al.* Scales to measure dimensions of hallucinations and delusions: The psychotic symptom rating scales (PSYRATS). *Psychol Med* 1999;**29**:879–89. doi:10.1017/s0033291799008661

25 Stefanis NC, Hanssen M, Smirnis NK, *et al.* Evidence that three dimensions of psychosis have a distribution in the general population. *Psychol Med* 2002;**32**:347–58. doi:10.1017/s0033291701005141

26 Kroenke K, Spitzer RL, Williams JB. The PHQ-9: validity of a brief depression severity measure. *J Gen Intern Med* 2001;**16**:606–13. doi:10.1046/j.1525-1497.2001.016009606.x

27 Spitzer RL, Kroenke K, Williams JBW, *et al.* A brief measure for assessing generalized anxiety disorder: the GAD-7. *Arch Intern Med* 2006;**166**:1092–7. doi:10.1001/archinte.166.10.1092

28 Mundt JC, Marks IM, Shear MK, *et al.* The Work and Social Adjustment Scale: a simple measure of impairment in functioning. *Br J Psychiatry* 2002;**180**:461–4. doi:10.1192/bjp.180.5.461

29 Herdman M, Gudex C, Lloyd A, *et al.* Development and preliminary testing of the new five-level version of EQ-5D (EQ-5D-5L). *Qual Life Res* 2011;**20**:1727–36. doi:10.1007/s11136-011-9903-x

30 Skinner HA. The drug abuse screening test. *Addict Behav* 1982;**7**:363–71. doi:10.1016/0306-4603(82)90005-3

31 Shapiro F. *Eye Movement Desensitization and Reprocessing (EMDR) Therapy: Basic Principles, Protocols and Procedures*. 3rd ed. New York, NY: : The Guilford Press 2018.

For peer review only

32 Ward-Brown J, Keane D, Bhutani G, *et al.* TF-CBT and EMDR for young people with trauma and first episode psychosis (using a phasic treatment approach): two early intervention service case studies. *Cogn Behav Ther* 2018;**11**:e17. doi:DOI: 10.1017/S1754470X18000193

33 Leeds A. *A guide to the standard EMDR therapy protocols for clinicians, supervisors, and consultants*. 2nd ed. New York: : Springer Publishing Company 2016.

34 Teare MD, Dimairo M, Shephard N, *et al.* Sample size requirements to estimate key design parameters from external pilot randomised controlled trials: a simulation study. *Trials* 2014;**15**:264. doi:10.1186/1745-6215-15-264

35 StataCorp. Stata Statistical Software: Release 15. 2017.

36 Sturges JE, Hanrahan KJ. Comparing Telephone and Face-to-Face Qualitative Interviewing: a Research Note. *Qual Res* 2004;**4**:107–18. doi:10.1177/1468794104041110

37 QSR International Pty Ltd. NVivo (Version 12). 2018.https://www.qsrinternational.com/nvivo-qualitative-data-analysis-software/home

38 Ritchie J, Spencer L. Analyzing Qualitative Data. In: Bryman A, Burgess R, eds. *Analyzing Qualitative Data*. London: 1994. 173–94.

39 Parry GD, Crawford MJ, Duggan C. Iatrogenic harm from psychological therapies-time to move on. *Br J Psychiatry* 2016;**208**:210–2. doi:10.1192/bjp.bp.115.163618

For peer review only

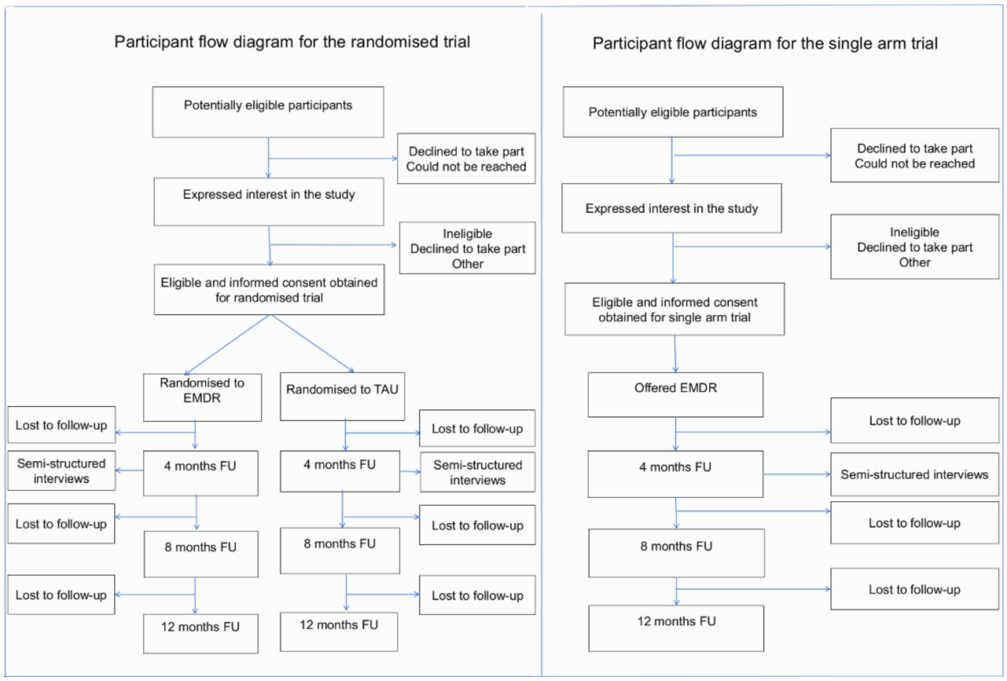

Figure 1. Study flow chart for the feasibility trial (randomised and non-randomised components)

89x60mm (400 x 400 DPI)

SPIRIT
STANDARD PROTOCOL ITEMS: RECOMMENDATIONS FOR INTERVENTIONAL TRIALS

SPIRIT 2013 Checklist: Recommended items to address in a clinical trial protocol and related documents*

Section/item	Item No	Description
**Administrative information**		
Title	1	Descriptive title identifying the study design, population, interventions, and, if applicable, trial acronym<br>- Page 1
Trial registration	2a	Trial identifier and registry name. If not yet registered, name of intended registry<br>- Page 2
	2b	All items from the World Health Organization Trial Registration Data Set<br>- Please see: https://www.isrctn.com/ISRCTN31976295?q=&filters=&sort=&offset=3&totalResults=16965&page=1&pageSize=10&searchType=basic-search
Protocol version	3	Date and version identifier<br>- Page 14
Funding	4	Sources and types of financial, material, and other support<br>- Page 14
Roles and responsibilities	5a	Names, affiliations, and roles of protocol contributors<br>- Page 1, 14
	5b	Name and contact information for the trial sponsor<br>- Page 14
	5c	Role of study sponsor and funders, if any, in study design; collection, management, analysis, and interpretation of data; writing of the report; and the decision to submit the report for publication, including whether they will have ultimate authority over any of these activities<br>- Page 14

For peer review only

	5d	Composition, roles, and responsibilities of the coordinating centre, steering committee, endpoint adjudication committee, data management team, and other individuals or groups overseeing the trial, if applicable (see Item 21a for data monitoring committee)<br>- Our Trial Steering Committee (TSC) has got three external members (trialist, psychosis expert and statistician). The role of the TSC is to provide oversight of the trial on behalf of the Trial Sponsor and funder. The main tasks of the TSC are: to provide expert advice on all aspects of the trial, to focus on the progress of the trial, adherence to the protocol, patient safety, to agree proposals for substantial protocol amendments, to monitor and review issues related to patient safety. The TSC will also take on data monitoring role for this feasibility study.
**Introduction**		
Background and rationale	6a	Description of research question and justification for undertaking the trial, including summary of relevant studies (published and unpublished) examining benefits and harms for each intervention<br>- Page 3-4
	6b	Explanation for choice of comparators<br>- Page 4, 7-8
Objectives	7	Specific objectives or hypotheses<br>- Page 4
Trial design	8	Description of trial design including type of trial (eg, parallel group, crossover, factorial, single group), allocation ratio, and framework (eg, superiority, equivalence, noninferiority, exploratory)<br>- Page 4-5
**Methods: Participants, interventions, and outcomes**		
Study setting	9	Description of study settings (eg, community clinic, academic hospital) and list of countries where data will be collected. Reference to where list of study sites can be obtained<br>- Page 5-6
Eligibility criteria	10	Inclusion and exclusion criteria for participants. If applicable, eligibility criteria for study centres and individuals who will perform the interventions (eg, surgeons, psychotherapists)<br>- Page 5
Interventions	11a	Interventions for each group with sufficient detail to allow replication, including how and when they will be administered<br>- Page 7-8

For peer review only

	11b	Criteria for discontinuing or modifying allocated interventions for a given trial participant (eg, drug dose change in response to harms, participant request, or improving/worsening disease)<br>- If a serious adverse event (SAE) occurs in relation to therapy, or participant has difficulties engaging with therapy to the point where therapy is no longer worthwhile, the therapy will be discontinued.
	11c	Strategies to improve adherence to intervention protocols, and any procedures for monitoring adherence (eg, drug tablet return, laboratory tests)<br>- For monitoring adherence, therapists in the study will inform members of the research team if participants have not attended therapy sessions.
	11d	Relevant concomitant care and interventions that are permitted or prohibited during the trial<br>- As mentioned in the exclusion criteria, participants receiving EMDR should not be currently receiving any other psychological therapy. Apart from this, participants in the trial can have access to any form of support/care that the NHS offers to patients at risk of psychosis.
Outcomes	12	Primary, secondary, and other outcomes, including the specific measurement variable (eg, systolic blood pressure), analysis metric (eg, change from baseline, final value, time to event), method of aggregation (eg, median, proportion), and time point for each outcome. Explanation of the clinical relevance of chosen efficacy and harm outcomes is strongly recommended<br>- Page 9
Participant timeline	13	Time schedule of enrolment, interventions (including any run-ins and washouts), assessments, and visits for participants. A schematic diagram is highly recommended (see Figure)<br>- See Figure 1.
Sample size	14	Estimated number of participants needed to achieve study objectives and how it was determined, including clinical and statistical assumptions supporting any sample size calculations<br>- Page 8-10
Recruitment	15	Strategies for achieving adequate participant enrolment to reach target sample size<br>- Page 5-6

Methods: Assignment of interventions (for controlled trials)

Allocation:

Sequence generation	16a	Method of generating the allocation sequence (eg, computer-generated random numbers), and list of any factors for stratification. To reduce predictability of a random sequence, details of any planned restriction (eg, blocking) should be provided in a separate document that is unavailable to those who enrol participants or assign interventions<br>- Page 8
Allocation concealment mechanism	16b	Mechanism of implementing the allocation sequence (eg, central telephone; sequentially numbered, opaque, sealed envelopes), describing any steps to conceal the sequence until interventions are assigned<br>- Page 8
Implementation	16c	Who will generate the allocation sequence, who will enrol participants, and who will assign participants to interventions<br>- Page 8
Blinding (masking)	17a	Who will be blinded after assignment to interventions (eg, trial participants, care providers, outcome assessors, data analysts), and how<br>- Page 8
	17b	If blinded, circumstances under which unblinding is permissible, and procedure for revealing a participant's allocated intervention during the trial<br>- Not applicable
**Methods: Data collection, management, and analysis**		
Data collection methods	18a	Plans for assessment and collection of outcome, baseline, and other trial data, including any related processes to promote data quality (eg, duplicate measurements, training of assessors) and a description of study instruments (eg, questionnaires, laboratory tests) along with their reliability and validity, if known. Reference to where data collection forms can be found, if not in the protocol<br>- Please see page 6-7 for key references to questionnaires
	18b	Plans to promote participant retention and complete follow-up, including list of any outcome data to be collected for participants who discontinue or deviate from intervention protocols<br>- If there are any concerns about complete data collection, research team will prioritise collection of data on psychotic symptoms, PTSD symptoms and medication (using the instruments described on page 9.

Data management	19	Plans for data entry, coding, security, and storage, including any related processes to promote data quality (eg, double data entry; range checks for data values). Reference to where details of data management procedures can be found, if not in the protocol<br>- Page 12
Statistical methods	20a	Statistical methods for analysing primary and secondary outcomes. Reference to where other details of the statistical analysis plan can be found, if not in the protocol<br>- Page 9-11
	20b	Methods for any additional analyses (eg, subgroup and adjusted analyses)<br>- Page 11
	20c	Definition of analysis population relating to protocol non-adherence (eg, as randomised analysis), and any statistical methods to handle missing data (eg, multiple imputation)<br>- Data from the randomised and single arm trial will be analysed as described on page 9-10
**Methods: Monitoring**		
Data monitoring	21a	Composition of data monitoring committee (DMC); summary of its role and reporting structure; statement of whether it is independent from the sponsor and competing interests; and reference to where further details about its charter can be found, if not in the protocol. Alternatively, an explanation of why a DMC is not needed<br>- As this is a small feasibility trial, the responsibilities of the DMC will be taken over by the TSC; composition, roles and responsibilities of the TSC can be seen at Item 5d.
	21b	Description of any interim analyses and stopping guidelines, including who will have access to these interim results and make the final decision to terminate the trial<br>- N/A.
Harms	22	Plans for collecting, assessing, reporting, and managing solicited and spontaneously reported adverse events and other unintended effects of trial interventions or trial conduct<br>- Page 13
Auditing	23	Frequency and procedures for auditing trial conduct, if any, and whether the process will be independent from investigators and the sponsor<br>- The decision to audit the trial rests with University Hospitals Bristol (UHB) which is independent from investigators and sponsor.

Ethics and dissemination

Research ethics approval	24	Plans for seeking research ethics committee/institutional review board (REC/IRB) approval<br>- Ethics approval received as described on page 15
Protocol amendments	25	Plans for communicating important protocol modifications (eg, changes to eligibility criteria, outcomes, analyses) to relevant parties (eg, investigators, REC/IRBs, trial participants, trial registries, journals, regulators)<br>- Amendments to the original protocol have been discussed with the TSC. All relevant parties, including the ISRCTN, have been notified of changes to the protocol.
Consent or assent	26a	Who will obtain informed consent or assent from potential trial participants or authorised surrogates, and how (see Item 32)<br>- Written informed consent will be obtained by researchers in the trial, as described on page 6-7.
	26b	Additional consent provisions for collection and use of participant data and biological specimens in ancillary studies, if applicable<br>- Not applicable
Confidentiality	27	How personal information about potential and enrolled participants will be collected, shared, and maintained in order to protect confidentiality before, during, and after the trial<br>- Page 12
Declaration of interests	28	Financial and other competing interests for principal investigators for the overall trial and each study site<br>- The authors declare no conflict of interests
Access to data	29	Statement of who will have access to the final trial dataset, and disclosure of contractual agreements that limit such access for investigators<br>- Only the immediate research team will have access to the final trial dataset.
Ancillary and post-trial care	30	Provisions, if any, for ancillary and post-trial care, and for compensation to those who suffer harm from trial participation<br>- There will be no post-trial care for participants who were randomised to treatment as usual as part of the original design of the study. This has been communicated to participants upon enrolling in the trial.
Dissemination policy	31a	Plans for investigators and sponsor to communicate trial results to participants, healthcare professionals, the public, and other relevant groups (eg, via publication, reporting in results databases, or other data sharing arrangements), including any publication restrictions<br>- Page 13

For peer review

	31b	Authorship eligibility guidelines and any intended use of professional writers<br>- Authorship will follow the ICMJE recommendations (substantial contribution to the design of the study, analysis or data interpretation, drafting or revising the work, final approval or the published version and agreement to be accountable for all aspects of the work).
	31c	Plans, if any, for granting public access to the full protocol, participant-level dataset, and statistical code<br>- The full protocol can be accessed online at https://www.isrctn.com/ISRCTN31976295?q=&filters=&sort=&offset=3&totalResults=16965&page=1&pageSize=10&searchType=basic-search<br>- Participants gave consent for their data to be anonymised and made available for use in future studies. Data will be available upon reasonable request via the first author.
**Appendices**		
Informed consent materials	32	Model consent form and other related documentation given to participants and authorised surrogates<br>- Please see the attached Participant Information Sheet
Biological specimens	33	Plans for collection, laboratory evaluation, and storage of biological specimens for genetic or molecular analysis in the current trial and for future use in ancillary studies, if applicable<br>- Not applicable

*It is strongly recommended that this checklist be read in conjunction with the SPIRIT 2013 Explanation & Elaboration for important clarification on the items. Amendments to the protocol should be tracked and dated. The SPIRIT checklist is copyrighted by the SPIRIT Group under the Creative Commons "Attribution-NonCommercial-NoDerivs 3.0 Unported" license.

For peer review only

A feasibility study of eye-movement desensitization and reprocessing (EMDR) in people with an at-risk mental state (ARMS) for psychosis: study protocol

Journal:	*BMJ Open*
Manuscript ID	bmjopen-2020-038620.R2
Article Type:	Protocol
Date Submitted by the Author:	19-Aug-2020
Complete List of Authors:	Strelchuk, Daniela; University of Bristol, Centre for Academic Mental Health, Population Health Sciences, Bristol Medical School; University Hospitals Bristol NHS Foundation Trust, National Institute for Health Research Bristol Biomedical Research Centre<br>Wiles, Nicola; University of Bristol, Centre for Academic Mental Health, Population Health Sciences, Bristol Medical School; University Hospitals Bristol NHS Foundation Trust, National Institute for Health Research Bristol Biomedical Research Centre<br>Turner, Katrina; University of Bristol, Centre for Academic Mental Health, Population Health Sciences, Bristol Medical School; University Hospitals Bristol NHS Foundation Trust, National Institute for Health Research Bristol Biomedical Research Centre<br>Derrick, Catherine; University of Bristol, Centre for Academic Mental Health, Population Health Sciences, Bristol Medical School<br>Zammit, Stan; University of Bristol, Centre for Academic Mental Health, Population Health Sciences, Bristol Medical School; Cardiff University, MRC Centre for Neuropsychiatric Genetics and Genomics, Division of Psychological Medicine and Clinical Neuroscience
**Primary Subject Heading**:	Mental health
Secondary Subject Heading:	Qualitative research, Evidence based practice
Keywords:	Schizophrenia & psychotic disorders < PSYCHIATRY, QUALITATIVE RESEARCH, Clinical trials < THERAPEUTICS

SCHOLARONE™
Manuscripts

I, the Submitting Author has the right to grant and does grant on behalf of all authors of the Work (as defined in the below author licence), an exclusive licence and/or a non-exclusive licence for contributions from authors who are: i) UK Crown employees; ii) where BMJ has agreed a CC-BY licence shall apply, and/or iii) in accordance with the terms applicable for US Federal Government officers or employees acting as part of their official duties; on a worldwide, perpetual, irrevocable, royalty-free basis to BMJ Publishing Group Ltd ("BMJ") its licensees and where the relevant Journal is co-owned by BMJ to the co-owners of the Journal, to publish the Work in this journal and any other BMJ products and to exploit all rights, as set out in our licence.

The Submitting Author accepts and understands that any supply made under these terms is made by BMJ to the Submitting Author unless you are acting as an employee on behalf of your employer or a postgraduate student of an affiliated institution which is paying any applicable article publishing charge ("APC") for Open Access articles. Where the Submitting Author wishes to make the Work available on an Open Access basis (and intends to pay the relevant APC), the terms of reuse of such Open Access shall be governed by a Creative Commons licence – details of these licences and which Creative Commons licence will apply to this Work are set out in our licence referred to above.

Other than as permitted in any relevant BMJ Author's Self Archiving Policies, I confirm this Work has not been accepted for publication elsewhere, is not being considered for publication elsewhere and does not duplicate material already published. I confirm all authors consent to publication of this Work and authorise the granting of this licence.

For peer review only

A feasibility study of eye-movement desensitization and reprocessing (EMDR) in people with an at-risk mental state (ARMS) for psychosis: study protocol

Daniela Strelchuk, MSc, Centre for Academic Mental Health, Population Health Sciences, Bristol Medical School, University of Bristol, Bristol, UK and National Institute for Health Research Bristol Biomedical Research Centre, University Hospitals Bristol NHS Foundation Trust and University of Bristol.

Nicola Wiles, PhD, Centre for Academic Mental Health, Population Health Sciences, Bristol Medical School, University of Bristol, Bristol, UK, and National Institute for Health Research Bristol Biomedical Research Centre, University Hospitals Bristol NHS Foundation Trust and University of Bristol.

Katrina Turner, PhD, Centre for Academic Mental Health, Population Health Sciences, Bristol Medical School, University of Bristol, Bristol, UK, and National Institute for Health Research Bristol Biomedical Research Centre, University Hospitals Bristol NHS Foundation Trust and University of Bristol.

Catherine Derrick, Centre for Academic Mental Health, Population Health Sciences, Bristol Medical School, University of Bristol, Bristol, UK

Stan Zammit, PhD, Centre for Academic Mental Health, Population Health Sciences, Bristol Medical School, University of Bristol, Bristol, UK; MRC Centre for Neuropsychiatric Genetics and Genomics, Division of Psychological Medicine and Clinical Neuroscience, Cardiff University, Cardiff, UK and National Institute for Health Research Bristol Biomedical Research Centre, University Hospitals Bristol NHS Foundation Trust and University of Bristol.

Corresponding author:

Daniela Strelchuk

Oakfield House, Oakfield Grove, Bristol, BS8 2BN;

Email: daniela.strelchuk@bristol.ac.uk

Tel: 07575540193

Word count: 3,958

For peer review only - http://bmjopen.bmj.com/site/about/guidelines.xhtml

Abstract

Introduction: Trauma can play an important role in the development of psychosis, yet no studies have investigated whether a trauma-focused psychological therapy could prevent the onset of psychosis in people at high risk of developing this condition. This study aims to establish whether it would be feasible to conduct a multi-centre randomised controlled trial (RCT) to investigate the clinical and cost effectiveness of eye movement desensitization and reprocessing therapy (EMDR) to prevent the onset of psychosis in people with an at-risk mental state (ARMS).

Methods/analysis: This is a single arm trial with a nested qualitative study where all participants (target n=20) will be offered EMDR. Eligible participants are those who meet criteria for ARMS; have experienced a traumatic event before the onset of ARMS symptomatology; and have at least one symptom of post-traumatic stress disorder (PTSD). Participants will be followed-up at 4, 8 and 12 months after the baseline assessment. The primary outcome measure is transition to psychosis, and secondary outcome measures include severity of psychotic symptoms, PTSD, depression, anxiety, impaired functioning, health status, and resource use. The analysis will aim to establish the rates of recruitment and retention for a large-scale RCT. Interviews with therapists and patients will explore their views of the study and their experiences of delivering or receiving EMDR.

Ethics and dissemination

This protocol has been approved by the South West-Cornwall & Plymouth Research Ethics Committee (Reference 18/SW/0037). Findings will be disseminated through journal publications, conference presentations and meetings with service users, their families, mental health professionals and commissioners.

Trial registration: ISRCTN31976295

For peer review only

Strengths and limitations of the study

- This is the first study to investigate the feasibility of conducting a large multi-centre RCT of EMDR for the prevention of psychosis in people with ARMS
- The study includes qualitative work to explore therapists' and patients' views of the treatment offered/received
- Therapy sessions follow an EMDR protocol specifically designed for people with psychosis
- The decision to change the study design to a single arm trial may limit our ability to draw robust conclusions about recruiting to an RCT

For peer review only - http://bmjopen.bmj.com/site/about/guidelines.xhtml

INTRODUCTION

Psychotic illnesses are one of the leading causes of disability worldwide.[1] The onset of psychosis is often preceded by a prodromal phase, known as an at-risk mental state (ARMS) for psychosis, which is characterized by a series of non-specific or attenuated psychotic symptoms.[2] Approximately 22% of ARMS individuals will transition to psychosis within one year and 36% of them within 3 years.[3]

Various interventions have been used for the prevention of psychosis, but there is still limited evidence on the effectiveness of these approaches. Clinical guidelines recommend cognitive-behavioural therapy (CBT) as a first-line treatment.[4] However, whilst a meta-analysis of randomized controlled trials showed that CBT has a moderate effect on transition to psychosis at 12 months follow-up (RR 0.64, 95% CI 0.44, 0.93), the evidence was of low to moderate quality and the effect was not maintained over the longer-term (18 months: RR 0.55, 95% CI 0.25, 1.19).[5]

Exposure to trauma is 2-3 times more common in ARMS or psychosis than in controls,[6–8] and post-traumatic stress disorder (PTSD) is also common, although not usually detected.[9] Despite strong evidence showing that trauma is a key factor in the development of psychosis,[10–12] no studies have yet investigated whether a trauma-focused therapy could prevent the onset of psychosis in ARMS.

Eye movement desensitisation and reprocessing (EMDR) is a trauma-focused therapy that has comparable efficacy to trauma-focused CBT (TF-CBT) in treating PTSD.[13–16] However, the two forms of therapy are different. While TF-CBT seeks to directly identify and restructure the negative beliefs, EMDR does this indirectly, relying more on the integration of emotional, cognitive, sensory, physical, and contextual information to allow adaptive processing of traumatic memories. Unlike TF-CBT, EMDR does not require patients to give a detailed description of the trauma, which may be distressing. Also, unlike TF-CBT, EMDR does not involve homework, which may make it more suitable for people with a disorganized lifestyle, such as those with ARMS.

To date, there has been only one randomised controlled trial (RCT) comparing EMDR with treatment as usual in patients with co-morbid psychosis and PTSD. This study showed that EMDR improved PTSD, and also reduced paranoid delusions, but did not reduce auditory hallucinations at 6 months follow-up.[17] However, the EMDR protocol did not specifically target psychotic symptoms. Furthermore, most participants had chronic forms of psychosis

For peer review only - http://bmjopen.bmj.com/site/about/guidelines.xhtml

and hence psychotic symptoms might have been less amenable to treatment than more recent onset symptoms present in ARMS subjects.

Objectives

The overarching aim of this study is to establish whether it would be feasible to conduct a large multi-centre RCT to evaluate the clinical and cost-effectiveness of EMDR to prevent the onset of psychosis in people with an at-risk mental state. The study has the following objectives:

1. To estimate the rate of recruitment and retention to inform the large-scale RCT
2. To refine the eligibility criteria, screening and recruitment procedures
3. To optimize the EMDR protocols and learn about the factors which affect the implementation of EMDR
4. To explore patients' and therapists' views of EMDR as a treatment for ARMS
5. To investigate patients' and therapists' views of the study design and study materials
6. To understand what treatment as usual (TAU) consists of for patients with ARMS

METHODS AND ANALYSIS

Study design

The study consists of a single arm feasibility trial with a nested qualitative study, and is conducted in collaboration with the Early Intervention (EI) teams from the Avon and Wiltshire Mental Health Partnership NHS Trust (AWP). All participants will be offered EMDR, and will be assessed at baseline, 4, 8 and 12 months follow-up as described below.

The study design outlined is the final version of the trial protocol. The original protocol was changed as a result of low recruitment. Recruitment challenges are described later.

Inclusion and exclusion criteria

Inclusion criteria

- Aged 16 years or over who are at risk of psychosis (as defined in the Comprehensive Assessment of At-Risk Mental States (CAARMS))[2]
- Presence of at least one positive symptom of psychosis (perceptual abnormality, unusual thought content, non-bizarre ideas or disorganised speech) scored ≥3 on CAARMS
- History of traumatic experience as defined in ICD-10 F43.1, occurring prior to onset of first positive symptom

For peer review only - http://bmjopen.bmj.com/site/about/guidelines.xhtml

For peer review only

- Presence of one or more symptoms of re-living, avoidance, hyper-arousal, or cognitive distortions in relation to the traumatic experience (assessed using the PTSD Checklist for DSM-V (PCL5)) during the last month.[18]

Exclusion criteria

- History of treated or untreated psychotic illness or learning disability
- Currently taking antipsychotics
- Currently receiving psychological therapy
- Completed a trauma-focused psychological therapy in the last 2 years
- Insufficient fluency in English
- Lacking mental capacity to provide valid informed consent

Recruitment procedure

Participants will be identified via two routes:

1. Early Intervention services

Researchers will identify clinical teams who are willing to identify potential participants via the following routes:

a) during routine clinic appointments - clinicians will introduce the study to patients who have met ARMS criteria within the past 12 months. Interested patients will be asked to complete section 1 of an Expression of Interest (EOI) form, and to provide their contact details. Completed EOI forms will be returned to the research team by secure email. A member of the research team will telephone interested individuals and, if the individual is willing to take part, agree a time and place for the researcher to meet with them to complete the eligibility assessment. Following the telephone call, the researcher will post the individual a full participant information sheet and a letter confirming the appointment.

Individuals who do not want to hear more about the study will be asked to complete section 2 of the EOI form, which asks for information on age, gender, reasons for non-participation and willingness to take part in a short interview (see ’qualitative data collection’ for more detail).

b) postal or telephone invitations from clinicians – If clinicians do not introduce the study to patients during the appointment, they will post ARMS patients a brief information sheet about the study, an EOI form and a stamped addressed envelope. Clinicians may also introduce the study to patients over the telephone. If the patient is interested, the clinician will ask the patient for permission to share their contact details with the research team. The clinician will then fill in the “Phone permission to contact” form and will email it to the research team. Recruitment

For peer review only - http://bmjopen.bmj.com/site/about/guidelines.xhtml

will then proceed as outlined in (a).

c) postal invitations sent out by NIHR CRN Clinical Studies Officers (CSO) - CSOs will pre-screen potential participants using the electronic patient record system (RIO), and liaise with the care team regarding potentially eligible patients who will then be sent a brief information sheet and details on how to contact the research team if they are interested in the study. Patients who do not reply will be followed up by telephone by the CSO within a week of receiving the documents. If the patient is interested in the study, the CSO will take permission over the phone to share their contact details with the research team. Once the research team have received contact details, recruitment will proceed as outlined in (a).

2. Everyone included within AWP

'Everyone Included' is a standard approach to research in AWP, whereby service users are routinely informed about relevant research opportunities. The AWP Research and Development department will send a 'Research Opportunity Letter' on behalf of the research team to potential participants briefly explaining the study and what it involves. A researcher will telephone individuals who are interested in the study and answer any questions. If the individual is willing to take part, an eligibility assessment will be arranged.

Consent and baseline eligibility assessment

Individuals interested in taking part will be invited to a face-to-face appointment with a researcher to establish eligibility, answer questions about participation, and obtain written informed consent.

The process of consenting young people (16 and 17 years old) will follow the Department of Health Reference Guide to Consent[19] and the Good Medical Practice Guidelines,[20] which provide the guide that all health professionals need to take into account in obtaining consent. Although young people may be more vulnerable than adults, they are presumed to be able to give consent to their treatment. As in the case of adults, if young people decline to take part in the study their decision will be fully respected and accepted.

In order to establish eligibility, patients will be asked to complete the following scales:

- Life-events Checklist for DSM-5 (LEC-5)[21]
- Childhood Trauma Questionnaire (CTQ)[22]
- PTSD Checklist for DSM-5 (PCL5)[18]

For peer review only - http://bmjopen.bmj.com/site/about/guidelines.xhtml

Those who are eligible will be asked to provide written informed consent for their participation in the trial and sociodemographic information. Additional baseline data will be collected using the following quantitative measures:

- Severity of psychotic symptoms: CAARMS,[2] the Negative Scale of the Positive and Negative Syndrome Scale (PANSS),[23] the Psychotic Symptom Rating Scales (PSYRAT)[24] and the Community Assessment of Psychic Experiences (CAPE-42).[25]
- Severity of depression and anxiety: Patient Health questionnaire (PHQ-9) for depression[26] and Generalized Anxiety Disorder Questionnaire (GAD-7) for anxiety[27]
- Impaired functioning: Work and Social Adjustment Scale (WSAS)[28]
- Health status: Five-level version of the EQ-5D (EQ-5D-5L)[29]
- Drug use: The Drug Abuse Screening Test (DAST 10)[30]

Data will also be collected on medication use via self-report measures. The baseline eligibility assessment will last about 90 minutes.

Intervention: EMDR

Participants will receive up to 12 sessions of manualized, weekly, face-to-face EMDR therapy.[31] Treatment has been manualized by EMDR consultants from Lancashire and South Cumbria Care NHS Foundation Trust (LSCFT) and individualised to target psychotic symptoms. The treatment protocol was developed and evaluated through pilot cases with first episode psychosis clients,[32] and further refined as part of an ongoing feasibility randomised controlled trial funded by the National Institute of Health Research (PB-PG-0317-20037; ISRCTN43816889). Therapeutic sessions will be delivered by EMDR UK & Europe trained therapists who will receive training based on the LSCFT manual for the study. The training will be tailored to therapists' needs, and will last between 4 to 8 hours. All EMDR therapists will have at least one year of experience of providing EMDR, and monthly supervision with an EMDR consultant will be in place. Each session will last approximately 90 minutes. The first 2-4 sessions will focus on establishing the therapeutic alliance, preparation for EMDR and stabilization techniques, assessment and identification of targets, cognitions, emotions and bodily sensations. The following 8-10 sessions will focus on desensitisation, installation of positive cognitions and body scan for past, present and future stressful situations.

Written permission will be sought to audio-record the therapeutic sessions. 10% of the sessions will be randomly sampled and evaluated by accredited EMDR therapists

For peer review only - http://bmjopen.bmj.com/site/about/guidelines.xhtml

independent of the study, to check treatment fidelity, using the EMDR fidelity checklist.[33] Therapists will be encouraged to liaise with participants' care coordinators as this could improve participants' adherence to therapy. If a serious adverse event (SAE) occurs in relation to therapy, or participant has difficulties engaging with therapy to the point where therapy is no longer worthwhile, the therapy will be discontinued.

Challenges to recruitment

The original feasibility study was designed as a RCT comparing EMDR with treatment as usual (TAU). TAU in AWP is quite heterogeneous and consists of discharge back to the referrer, signposting to other services, CBT, family intervention or psycho-education. Randomisation took place using a remote computerised randomisation service administered by the Bristol Trials Centre ensuring that allocations were concealed from the recruiting researcher. Randomisation was minimized by psychotic symptom severity (score on positive symptoms on CAARMS <11 or ≥11). However, following challenges to recruitment (described below), the study design was changed to a single arm study.

During the first seven months of the study (May to December 2018), we only recruited three participants (against a target of 21). This poor recruitment was primarily because the number of patients identified as ARMS by the EI teams within AWP was much lower than expected, and only three of the six EI teams we originally planned to recruit from were able to support the study due to financial constraints to managing ARMS clients. Therefore, from July 2019, all participants recruited to the study were offered EMDR. Figure 1 shows the flow of participants in the randomised and single arm phases of the trial.

Sample size and feasibility of recruitment

In the original protocol we had planned to recruit up to 40 participants. The initial sample size was informed by literature guidelines[34] and pragmatic considerations concerning potential difficulties accessing this population. The feasibility study would not be powered to detect important clinical differences between EMDR and TAU groups but would provide estimates of the completion and retention rates that would further assist in planning the recruitment for a future RCT.

Given the challenges with recruitment and modification of our study design (from a randomised to single arm trial), we decreased our target sample size from 40 to 20 participants. This sample size will provide us with reasonable precision around estimates of percentages completing the intervention and follow-ups. For example, if 70% of those who started EMDR complete the intervention, the 95% confidence intervals would be 45% and 88%. Similarly, if 75% of those who started EMDR are followed up to 12 months, the 95% confidence intervals

For peer review only - http://bmjopen.bmj.com/site/about/guidelines.xhtml

for the retention rate would be 50% and 91%.

Outcome assessment

The follow-up schedule will involve face-to-face assessments at 4, 8 and 12 months after the baseline assessment. Each follow-up assessment will last about 60 minutes. The primary outcome will be collected at 12 months follow-up, and the secondary outcome at 4, 8 and 12 months.

A 12-month follow-up will provide us with adequate data on retention rate that can be used to ensure that a definitive trial is adequately powered. If, in the definitive trial, the intervention was shown to be effective at 12 months, then further follow-up could examine whether effects are maintained over the longer-term.

Primary outcome

- Transition to psychosis: ICD-10 diagnosis of psychotic disorder from clinical records or via the CAARMS

Secondary outcome

- Severity of psychotic symptoms: CAARMS, PSYRAT, the negative scale of the PANSS and CAPE-42
- Severity of PTSD symptoms: PCL-5
- Severity of depression and anxiety: PHQ-9 and GAD-7
- Impaired functioning: WSAS
- Health status: EQ-5D-L
- Drug use: DAST 10
- Medication use
- Resource data use - we will ask questions about resource use including information on the use of primary and secondary care services, use of social services and disability payments received, time off school or work, and productivity loss due to time off work or study.

If there are difficulties following up trial participants to obtain outcome data, researchers will prioritise collection of data on psychotic symptoms, PTSD symptoms and medication. Following the switch to single arm design, it will no longer be possible to blind researchers to treatment allocation in terms of avoiding potential bias in outcome measurement.

For peer review only - http://bmjopen.bmj.com/site/about/guidelines.xhtml

Quantitative data analysis

Quantitative data will be analysed in Stata version 15.[35] If there are no differences in baseline characteristics of those who were randomised and those who enter the single arm study, we will combine data to generate summary statistics on: (1) the proportion consenting to take part in the study; (2) the proportion completing the baseline assessment and agreeing to take part in the study; (3) the number of EMDR sessions attended and the proportion completing 8 or more sessions (regarded as an adequate dose of therapy); (4) the proportion completing the follow-up assessments. Feasibility outcomes such as recruitment and retention rates will be calculated with 95% confidence intervals using the exact binomial method.

We will also report the proportion of individuals who transitioned to psychosis at 12 months, and the confidence intervals for the effect size will be calculated to assist the planning of a future RCT. We will also compare the continuous outcomes such as the severity of psychotic symptoms, depression, anxiety, PTSD symptoms and quality of life to show the frequency of data completion, mean and standard deviation for the three time points.

Qualitative data collection

We will conduct in-depth individual interviews with all the therapists and participants who participate in the trial. In addition, up to 10 individuals who declined to take part in the trial will be interviewed to explore their views and understanding of the study and EMDR, and their reasons for declining to take part.

To encourage participation, and because well-planned interviews can gather the same material as those conducted face-to-face,[36] therapists and study participants will be given the choice of being interviewed in person or over the telephone. Therapists will be interviewed within a month of finishing intervention delivery. The interviews will explore therapist views and experiences of delivering EMDR to patients with ARMS, how treatment could be better tailored to this patient group, and what resources they would need if the intervention was evaluated in a large scale RCT. The interviews will also be used to explore their views of the training and supervision received.

Interviews with participants who completed treatment or stopped early will explore their views and experiences of the treatment received, identify how treatment delivery could be refined to increase its acceptability, and how study materials could be improved. Participants who completed the study will be interviewed within a month of completing their 4-month follow-up, and those who stopped early will be interviewed within a month of doing so.

For peer review only - http://bmjopen.bmj.com/site/about/guidelines.xhtml

Topic guides will be developed for each set of interviews to ensure consistency across data collection. The therapist and study participant guides will be developed in parallel to ensure key areas are discussed with both practitioners and patients. This will help compare findings across the interviews, highlighting similarities and differences between the views and experiences of patients and practitioners, and increasing the confidence with which study conclusions can be drawn. With interviewee consent, the interviews will be audio-recorded and transcribed verbatim.

Qualitative data analysis

All interviews will be fully transcribed and anonymised. Data collection and analysis will proceed in parallel, so that insights from earlier data can shape later data collection, and enable the team to establish when data saturation has been reached. The data gathered will be analysed thematically. Transcripts will be independently coded by different researchers, who will then meet to discuss their coding and interpretation of the data. This will help to control for researcher bias and may lead to the coding frame being revised or defined more clearly. Transcripts will then be imported into the software package NVivo version 12[37] to allow electronic coding and retrieval of data. Once all the transcripts have been coded, data will be analysed using an approach similar to framework analysis.[38]

Additional qualitative work to address issues with recruitment and explore treatment options in ARMS populations

As the study progressed, to explore why recruitment had been so low, additional objectives were incorporated. These were:

1) To explore how potential ARMS patients are identified and managed in primary and secondary care services
2) To identify referral routes between primary and secondary care
3) To explore other researchers' experiences of recruiting ARMS patients to research studies in the UK

This additional qualitative work will entail:

a. Interviews with GPs, clinicians from Primary Care Liaison Services (PCLS) and Early Intervention services
b. Interviews with patients who did not participate in the interventional part of the study but who have been identified as ARMS by the EI teams we are recruiting from
c. Interviews with researchers who have been involved in recruiting ARMS patients

For peer review only - http://bmjopen.bmj.com/site/about/guidelines.xhtml

to research studies in the UK.

Summary of changes to the original feasibility study

There have been a number of changes to the original feasibility study. These are summarized below:

a) Change of study design from randomized trial to single arm trial
b) Decrease in sample size from 40 to 20 participants
c) Change in the measure used to assess the severity of psychotic symptoms at baseline and follow-up assessments from the Positive Scale of the PANSS to the CAARMS, as the latter is more sensitive
d) Extending the time between patients being identified as ARMS to the time when they can be recruited into the study from 3 to 12 months
e) Additional interviews with GPs, other clinicians from primary and secondary care services, ARMS patients and researchers to better understand identification and management of ARMS patients, including routes into primary and secondary services.

Study management

Data management

Completed questionnaires will be stored securely in compliance with University of Bristol Data Security policies and the General Data Protection Regulation (GDPR). Personal details will be entered onto a secure database held on the University of Bristol server, and non-identifiable data will be entered onto a secure web-based database. Data collected on paper will be identifiable only by Patient Identification Number (PIN). Information capable of identifying individuals will be held on the database with passwords restricted to authorised study staff only.

Data will be stored in the University of Bristol research data repository, and will be available upon reasonable request.

Patient and public involvement

Public and patient involvement (PPI) played an important role in this study. This research idea was initially presented at a PPI event which brought together people with lived experiences of psychosis, their carers, clinicians, academics and commissioners. Further on, this idea was presented at the Hearing Voices Network, and through these contacts we established a PPI group who gave us important feedback on the research question, the intervention, and which measures to use. We anticipate that the group, which is still active, will: (i) ensure that the

For peer review only - http://bmjopen.bmj.com/site/about/guidelines.xhtml

For peer review only

views of patients who have had a psychotic illness are heard at every stage of the study; (ii) inform any developments of the intervention to make sure these are acceptable to the target population, (iii) ensure patient documentation is easy to understand to maximise response and retention rates; and (iv) help interpret and disseminate study findings.

MONITORING AND ADVERSE EVENTS

Adverse events

We use the recommendations for defining and reporting adverse events and harm as outlined by Parry, Crawford, & Duggan (2016).[39] All serious adverse events will be reported to the Chief Investigator and the Trial Steering Committee (TSC). All SAEs of a related and unexpected nature will be reported to the main REC, in accordance with any procedures of the Sponsor. The decision to audit the trial rests with University Hospitals Bristol.

Trial oversight

A trial steering committee (TSC) will have independent oversight of the study, meeting at six monthly intervals during the course of the trial. The TSC will also take on data monitoring role for this feasibility study.

DISCUSSION

To our knowledge, this is the first study to investigate the feasibility of conducting a large RCT to establish whether EMDR could prevent the onset of psychosis in people with an at-risk mental state. As psychotic illnesses usually occur in early adulthood and disrupt a key period of professional and social development, preventing psychosis would improve patients' outcomes, and also decrease the substantial economic burden of this condition.

The results of this feasibility trial will provide us with a better understanding of this patient group, and will provide us with information that will be used to inform whether progression to a full trial would be feasible. The nested qualitative study will address issues of patient and therapist views of the intervention, and provide new insights into treatment pathways and experiences of this patient group. If shown to be feasible, a full scale RCT will be conducted to examine the clinical and cost-effectiveness of EMDR to prevent the onset of psychosis in ARMS.

For peer review only

For peer review only - http://bmjopen.bmj.com/site/about/guidelines.xhtml

ETHICS AND DISSEMINATION

This protocol has been approved by South West-Cornwall & Plymouth Research Ethics Committee (formerly South West-Exeter Research Ethics Committee), Reference 18/SW/0037, 29.03.2018.

The results of this study will be disseminated through publications and conferences. Findings from this feasibility trial will be published in psychiatry journals compliant with NIHR Open Access policy, and presented to clinical staff, service users, and relevant Clinical Commissioning Groups.

Feasibility trial status

Recruitment to this study opened in May 2018, and closed in May 2020. At the time of writing (July 2020), 14 participants have been recruited to the study. We expect to finish follow-up data collection by May 2021.

This article describes protocol version 4, dated 2.08.2019. When all regulatory approvals had been received, the protocol was version 1, dated 11.01.2018. Since then, two major amendments have been made with changes to incorporate additional qualitative work, decrease the sample size, and change the study design from a RCT to a single arm trial. The latter change took place in July 2019 prior to which we had recruited 6 participants.

Acknowledgements

We would like to thank Professor Jonathan Bisson, Professor Peter Jones and Dr Fiona Warren for overseeing the trial as part of our TSC group. The authors would also like to thank members of our PPI group Mr Bradley Jones, Ms Claire Barnard and Joanna Gigon for their feedback on the research question, intervention and measures to use, comments on the supporting documentation, and suggestions on how to improve recruitment and increase retention to the study.

Contributors

SZ, NW, KT and DS designed the study. SZ is the grant holder and Chief Investigator. SZ, NW and KT will oversee the scientific rigour of the study. NW will provide expertise in the management and analysis of the study. KT will oversee the nested qualitative study. DS will coordinate, implement and run the trial. CD will assist with running the trial. All authors contributed to refinement of the study protocol and approved the final manuscript.

For peer review only - http://bmjopen.bmj.com/site/about/guidelines.xhtml

Funding

This study was funded by the NIHR Biomedical Research Centre at University Hospitals Bristol NHS Foundation Trust (BRC-1215-20011) and the University of Bristol. The views expressed in this publication are those of the author(s) and not necessarily those of the NHS, the National Institute for Health Research or the Department of Health. The sponsor of the study is the University of Bristol. The sponsor and study funders had no authority over the running of the trial, and all decisions rest with the trial team.

Competing interests

The authors declare no conflict of interests

For peer review only

Legend

Figure 1. Study flow chart for the feasibility trial (randomised and non-randomised components)

REFERENCES

1 Vos T, Abajobir AA, Abbafati C, *et al.* Global, regional, and national incidence, prevalence, and years lived with disability for 328 diseases and injuries for 195 countries, 1990-2016: A systematic analysis for the Global Burden of Disease Study 2016. *Lancet* 2017;**390**:1211–59. doi:10.1016/S0140-6736(17)32154-2

2 Yung AR, Yuen HP, McGorry PD, *et al.* Mapping the onset of psychosis: the Comprehensive Assessment of At-Risk Mental States. *Aust N Z J Psychiatry* 2005;**39**:964–71. doi:10.1111/j.1440-1614.2005.01714.x

3 Fusar-Poli P. Predicting Psychosis. *Arch Gen Psychiatry* 2012;**69**:220. doi:10.1001/archgenpsychiatry.2011.1472

4 National Institute for Health and Care Excellence [NICE]. Psychosis and schizophrenia in adults: prevention and management. Retrieved from https://www.nice.org.uk/guidance/cg178/chapter/1-recommendations#preventing-psychosis-2. Retrieved from https//www.nice.org.uk/guidance/cg178/chapter/1-recommendations#preventing-psychosis-2 (accessed Jan 2020). 2014.

5 Stafford MR, Jackson H, Mayo-Wilson E, *et al.* Early interventions to prevent psychosis: Systematic review and meta-analysis. *BMJ* 2013;**346**:1–13. doi:10.1136/bmj.f185

6 Varese F, Smeets F, Drukker M, *et al.* Childhood adversities increase the risk of

For peer review only - http://bmjopen.bmj.com/site/about/guidelines.xhtml

psychosis: A meta-analysis of patient-control, prospective-and cross-sectional cohort studies. *Schizophr Bull* 2012;**38**:661–71. doi:10.1093/schbul/sbs050

7 Kraan T, Velthorst E, Smit F, *et al.* Trauma and recent life events in individuals at ultra high risk for psychosis: Review and meta-analysis. *Schizophr Res* 2015;**161**:143–9. doi:10.1016/j.schres.2014.11.026

8 Matheson SL, Shepherd AM, Pinchbeck RM, *et al.* Childhood adversity in schizophrenia: A systematic meta-analysis. *Psychol Med* 2013;**43**:225–38. doi:10.1017/S0033291712000785

9 Zammit S, Lewis C, Dawson S, *et al.* Undetected post-traumatic stress disorder in secondary-care mental health services: Systematic review. *Br J Psychiatry* 2018;**212**:11–8. doi:10.1192/bjp.2017.8

10 Trotta A, Murray RM, Fisher HL. The impact of childhood adversity on the persistence of psychotic symptoms: a systematic review and meta-analysis. *Psychol Med* 2015;**45**:2481–98. doi:10.1017/S0033291715000574

11 Van Dam DS, Van Der Ven E, Velthorst E, *et al.* Childhood bullying and the association with psychosis in non-clinical and clinical samples: A review and meta-analysis. *Psychol Med* 2012;**42**:2463–76. doi:10.1017/S0033291712000360

12 Croft J, Heron J, Teufel C, *et al.* Association of Trauma Type, Age of Exposure, and Frequency in Childhood and Adolescence with Psychotic Experiences in Early Adulthood. *JAMA Psychiatry* 2019;**76**:79–86. doi:10.1001/jamapsychiatry.2018.3155

13 Chen L, Zhang G, Hu M, *et al.* Eye movement desensitization and reprocessing versus cognitive-behavioral therapy for adult posttraumatic stress disorder: Systematic review and meta-analysis. *J Nerv Ment Dis* 2015;**203**:443–51. doi:10.1097/NMD.0000000000000306

14 Bisson J, Andrew M. Psychological treatment of post-traumatic stress disorder (PTSD). *Cochrane database Syst Rev* 2007;:CD003388. doi:10.1002/14651858.CD003388.pub3

15 Seidler GH, Wagner FE. Comparing the efficacy of EMDR and trauma-focused cognitive-behavioral therapy in the treatment of PTSD: A meta-analytic study. *Psychol Med* 2006;**36**:1515–22. doi:10.1017/S0033291706007963

16 Watts B V., Schnurr PP, Mayo L, *et al.* Meta-analysis of the efficacy of treatments for posttraumatic stress disorder. *J Clin Psychiatry* 2013;**74**:541–50. doi:10.4088/JCP.12r08225

17 de Bont PAJMJM, van den Berg DPGG, Van Der Vleugel BM, *et al.* Prolonged

For peer review only - http://bmjopen.bmj.com/site/about/guidelines.xhtml

exposure and EMDR for PTSD v. a PTSD waiting-list condition: effects on symptoms of psychosis, depression and social functioning in patients with chronic psychotic disorders. *Psychol Med* 2016;**46**:2411–21. doi:DOI: 10.1017/S0033291716001094

18 Bovin MJ, Marx BP, Weathers FW, *et al.* Psychometric properties of the PTSD Checklist for Diagnostic and Statistical Manual of Mental Disorders-Fifth Edition (PCL-5) in veterans. *Psychol Assess* 2016;**28**:1379–91. doi:10.1037/pas0000254

19 Department of Health and Social Care. Reference guide to consent for examination or treatment (second edition). 2009.https://www.gov.uk/government/publications/reference-guide-to-consent-for-examination-or-treatment-second-edition (accessed 3 May 2020).

20 General Medical Council. https://www.gmc-uk.org/static/documents/content/Good_medical_practice_-_English_1215.pdf. 2013.

21 Gray MJ, Litz BT, Hsu JL, *et al.* Psychometric properties of the life events checklist. *Assessment* 2004;**11**:330–41. doi:10.1177/1073191104269954

22 Bernstein DP, Fink L. *Childhood trauma questionnaire : a retrospective self-report : manual*. Orlando: : Psychological Corporation 1998.

23 Kay SR, Fiszbein A, Opler LA. The positive and negative syndrome scale (PANSS) for schizophrenia. *Schizophr Bull* 1987;**13**:261–76. doi:10.1093/schbul/13.2.261

24 Haddock G, McCarron J, Tarrier N, *et al.* Scales to measure dimensions of hallucinations and delusions: The psychotic symptom rating scales (PSYRATS). *Psychol Med* 1999;**29**:879–89. doi:10.1017/s0033291799008661

25 Stefanis NC, Hanssen M, Smirnis NK, *et al.* Evidence that three dimensions of psychosis have a distribution in the general population. *Psychol Med* 2002;**32**:347–58. doi:10.1017/s0033291701005141

26 Kroenke K, Spitzer RL, Williams JB. The PHQ-9: validity of a brief depression severity measure. *J Gen Intern Med* 2001;**16**:606–13. doi:10.1046/j.1525-1497.2001.016009606.x

27 Spitzer RL, Kroenke K, Williams JBW, *et al.* A brief measure for assessing generalized anxiety disorder: the GAD-7. *Arch Intern Med* 2006;**166**:1092–7. doi:10.1001/archinte.166.10.1092

28 Mundt JC, Marks IM, Shear MK, *et al.* The Work and Social Adjustment Scale: a simple measure of impairment in functioning. *Br J Psychiatry* 2002;**180**:461–4. doi:10.1192/bjp.180.5.461

29 Herdman M, Gudex C, Lloyd A, *et al.* Development and preliminary testing of the new

For peer review only - http://bmjopen.bmj.com/site/about/guidelines.xhtml

five-level version of EQ-5D (EQ-5D-5L). *Qual Life Res* 2011;**20**:1727–36. doi:10.1007/s11136-011-9903-x

30 Skinner HA. The drug abuse screening test. *Addict Behav* 1982;**7**:363–71. doi:10.1016/0306-4603(82)90005-3

31 Shapiro F. *Eye Movement Desensitization and Reprocessing (EMDR) Therapy: Basic Principles, Protocols and Procedures*. 3rd ed. New York, NY: : The Guilford Press 2018.

32 Ward-Brown J, Keane D, Bhutani G, *et al.* TF-CBT and EMDR for young people with trauma and first episode psychosis (using a phasic treatment approach): two early intervention service case studies. *Cogn Behav Ther* 2018;**11**:e17. doi:DOI: 10.1017/S1754470X18000193

33 Leeds A. *A guide to the standard EMDR therapy protocols for clinicians, supervisors, and consultants*. 2nd ed. New York: : Springer Publishing Company 2016.

34 Teare MD, Dimairo M, Shephard N, *et al.* Sample size requirements to estimate key design parameters from external pilot randomised controlled trials: a simulation study. *Trials* 2014;**15**:264. doi:10.1186/1745-6215-15-264

35 StataCorp. Stata Statistical Software: Release 15. 2017.

36 Sturges JE, Hanrahan KJ. Comparing Telephone and Face-to-Face Qualitative Interviewing: a Research Note. *Qual Res* 2004;**4**:107–18. doi:10.1177/1468794104041110

37 QSR International Pty Ltd. NVivo (Version 12). 2018.https://www.qsrinternational.com/nvivo-qualitative-data-analysis-software/home

38 Ritchie J, Spencer L. Analyzing Qualitative Data. In: Bryman A, Burgess R, eds. *Analyzing Qualitative Data*. London: 1994. 173–94.

39 Parry GD, Crawford MJ, Duggan C. Iatrogenic harm from psychological therapies-time to move on. *Br J Psychiatry* 2016;**208**:210–2. doi:10.1192/bjp.bp.115.163618

For peer review only

For peer review only

For peer review only - http://bmjopen.bmj.com/site/about/guidelines.xhtml

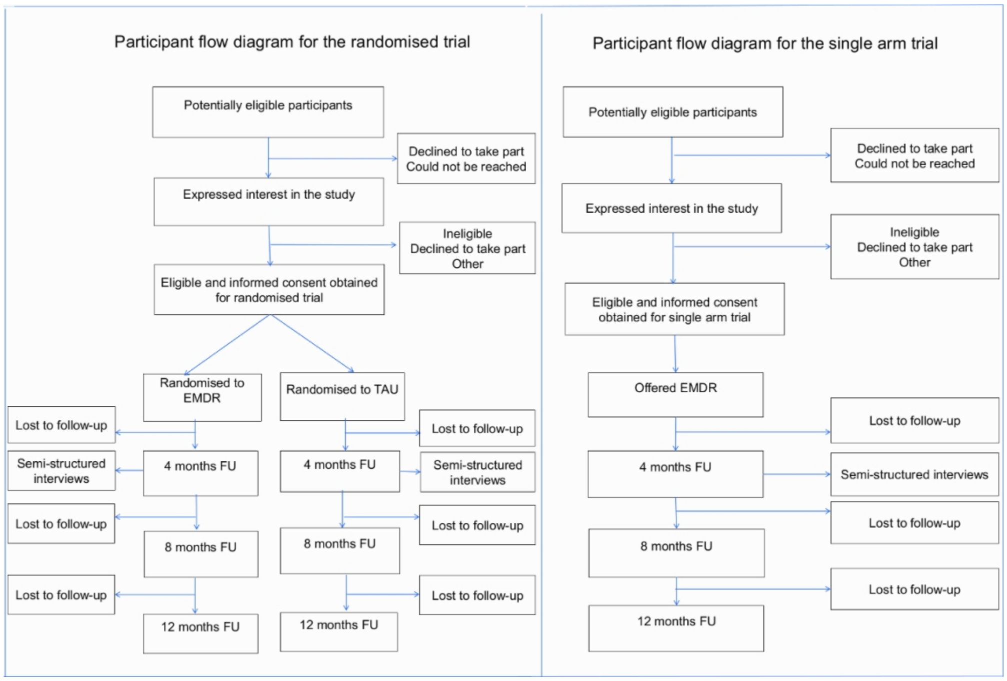

Figure 1. Study flow chart for the feasibility trial (randomised and non-randomised components)

89x60mm (400 x 400 DPI)

SPIRIT

STANDARD PROTOCOL ITEMS: RECOMMENDATIONS FOR INTERVENTIONAL TRIALS

SPIRIT 2013 Checklist: Recommended items to address in a clinical trial protocol and related documents*

Section/item	Item No	Description
**Administrative information**		
Title	1	Descriptive title identifying the study design, population, interventions, and, if applicable, trial acronym<br>- Page 1
Trial registration	2a	Trial identifier and registry name. If not yet registered, name of intended registry<br>- Page 2
	2b	All items from the World Health Organization Trial Registration Data Set<br>- Please see: https://www.isrctn.com/ISRCTN31976295?q=&filters=&sort=&offset=3&totalResults=16965&page=1&pageSize=10&searchType=basic-search
Protocol version	3	Date and version identifier<br>- Page 14
Funding	4	Sources and types of financial, material, and other support<br>- Page 14
Roles and responsibilities	5a	Names, affiliations, and roles of protocol contributors<br>- Page 1, 14
	5b	Name and contact information for the trial sponsor<br>- Page 14
	5c	Role of study sponsor and funders, if any, in study design; collection, management, analysis, and interpretation of data; writing of the report; and the decision to submit the report for publication, including whether they will have ultimate authority over any of these activities<br>- Page 14

For peer review only - http://bmjopen.bmj.com/site/about/guidelines.xhtml

	5d	Composition, roles, and responsibilities of the coordinating centre, steering committee, endpoint adjudication committee, data management team, and other individuals or groups overseeing the trial, if applicable (see Item 21a for data monitoring committee)<br>- Our Trial Steering Committee (TSC) has got three external members (trialist, psychosis expert and statistician). The role of the TSC is to provide oversight of the trial on behalf of the Trial Sponsor and funder. The main tasks of the TSC are: to provide expert advice on all aspects of the trial, to focus on the progress of the trial, adherence to the protocol, patient safety, to agree proposals for substantial protocol amendments, to monitor and review issues related to patient safety. The TSC will also take on data monitoring role for this feasibility study.
**Introduction**		
Background and rationale	6a	Description of research question and justification for undertaking the trial, including summary of relevant studies (published and unpublished) examining benefits and harms for each intervention<br>- Page 3-4
	6b	Explanation for choice of comparators<br>- Page 4, 7-8
Objectives	7	Specific objectives or hypotheses<br>- Page 4
Trial design	8	Description of trial design including type of trial (eg, parallel group, crossover, factorial, single group), allocation ratio, and framework (eg, superiority, equivalence, noninferiority, exploratory)<br>- Page 4-5
**Methods: Participants, interventions, and outcomes**		
Study setting	9	Description of study settings (eg, community clinic, academic hospital) and list of countries where data will be collected. Reference to where list of study sites can be obtained<br>- Page 5-6
Eligibility criteria	10	Inclusion and exclusion criteria for participants. If applicable, eligibility criteria for study centres and individuals who will perform the interventions (eg, surgeons, psychotherapists)<br>- Page 5
Interventions	11a	Interventions for each group with sufficient detail to allow replication, including how and when they will be administered<br>- Page 7-8

For peer review only

	11b	Criteria for discontinuing or modifying allocated interventions for a given trial participant (eg, drug dose change in response to harms, participant request, or improving/worsening disease)<br>- If a serious adverse event (SAE) occurs in relation to therapy, or participant has difficulties engaging with therapy to the point where therapy is no longer worthwhile, the therapy will be discontinued.
	11c	Strategies to improve adherence to intervention protocols, and any procedures for monitoring adherence (eg, drug tablet return, laboratory tests)<br>- For monitoring adherence, therapists in the study will inform members of the research team if participants have not attended therapy sessions.
	11d	Relevant concomitant care and interventions that are permitted or prohibited during the trial<br>- As mentioned in the exclusion criteria, participants receiving EMDR should not be currently receiving any other psychological therapy. Apart from this, participants in the trial can have access to any form of support/care that the NHS offers to patients at risk of psychosis.
Outcomes	12	Primary, secondary, and other outcomes, including the specific measurement variable (eg, systolic blood pressure), analysis metric (eg, change from baseline, final value, time to event), method of aggregation (eg, median, proportion), and time point for each outcome. Explanation of the clinical relevance of chosen efficacy and harm outcomes is strongly recommended<br>- Page 9
Participant timeline	13	Time schedule of enrolment, interventions (including any run-ins and washouts), assessments, and visits for participants. A schematic diagram is highly recommended (see Figure)<br>- See Figure 1.
Sample size	14	Estimated number of participants needed to achieve study objectives and how it was determined, including clinical and statistical assumptions supporting any sample size calculations<br>- Page 8-10
Recruitment	15	Strategies for achieving adequate participant enrolment to reach target sample size<br>- Page 5-6

Methods: Assignment of interventions (for controlled trials)

Allocation:

For peer review only - http://bmjopen.bmj.com/site/about/guidelines.xhtml

Sequence generation	16a	Method of generating the allocation sequence (eg, computer-generated random numbers), and list of any factors for stratification. To reduce predictability of a random sequence, details of any planned restriction (eg, blocking) should be provided in a separate document that is unavailable to those who enrol participants or assign interventions<br>- Page 8
Allocation concealment mechanism	16b	Mechanism of implementing the allocation sequence (eg, central telephone; sequentially numbered, opaque, sealed envelopes), describing any steps to conceal the sequence until interventions are assigned<br>- Page 8
Implementation	16c	Who will generate the allocation sequence, who will enrol participants, and who will assign participants to interventions<br>- Page 8
Blinding (masking)	17a	Who will be blinded after assignment to interventions (eg, trial participants, care providers, outcome assessors, data analysts), and how<br>- Page 8
	17b	If blinded, circumstances under which unblinding is permissible, and procedure for revealing a participant's allocated intervention during the trial<br>- Not applicable

Methods: Data collection, management, and analysis

Data collection methods	18a	Plans for assessment and collection of outcome, baseline, and other trial data, including any related processes to promote data quality (eg, duplicate measurements, training of assessors) and a description of study instruments (eg, questionnaires, laboratory tests) along with their reliability and validity, if known. Reference to where data collection forms can be found, if not in the protocol<br>- Please see page 6-7 for key references to questionnaires
	18b	Plans to promote participant retention and complete follow-up, including list of any outcome data to be collected for participants who discontinue or deviate from intervention protocols<br>- If there are any concerns about complete data collection, research team will prioritise collection of data on psychotic symptoms, PTSD symptoms and medication (using the instruments described on page 9.

Data management	19	Plans for data entry, coding, security, and storage, including any related processes to promote data quality (eg, double data entry; range checks for data values). Reference to where details of data management procedures can be found, if not in the protocol<br>- Page 12
Statistical methods	20a	Statistical methods for analysing primary and secondary outcomes. Reference to where other details of the statistical analysis plan can be found, if not in the protocol<br>- Page 9-11
	20b	Methods for any additional analyses (eg, subgroup and adjusted analyses)<br>- Page 11
	20c	Definition of analysis population relating to protocol non-adherence (eg, as randomised analysis), and any statistical methods to handle missing data (eg, multiple imputation)<br>- Data from the randomised and single arm trial will be analysed as described on page 9-10
**Methods: Monitoring**		
Data monitoring	21a	Composition of data monitoring committee (DMC); summary of its role and reporting structure; statement of whether it is independent from the sponsor and competing interests; and reference to where further details about its charter can be found, if not in the protocol. Alternatively, an explanation of why a DMC is not needed<br>- As this is a small feasibility trial, the responsibilities of the DMC will be taken over by the TSC; composition, roles and responsibilities of the TSC can be seen at Item 5d.
	21b	Description of any interim analyses and stopping guidelines, including who will have access to these interim results and make the final decision to terminate the trial<br>- N/A.
Harms	22	Plans for collecting, assessing, reporting, and managing solicited and spontaneously reported adverse events and other unintended effects of trial interventions or trial conduct<br>- Page 13
Auditing	23	Frequency and procedures for auditing trial conduct, if any, and whether the process will be independent from investigators and the sponsor<br>- The decision to audit the trial rests with University Hospitals Bristol (UHB) which is independent from investigators and sponsor.

For peer review only - http://bmjopen.bmj.com/site/about/guidelines.xhtml

Ethics and dissemination

Research ethics approval	24	Plans for seeking research ethics committee/institutional review board (REC/IRB) approval<br>- Ethics approval received as described on page 15
Protocol amendments	25	Plans for communicating important protocol modifications (eg, changes to eligibility criteria, outcomes, analyses) to relevant parties (eg, investigators, REC/IRBs, trial participants, trial registries, journals, regulators)<br>- Amendments to the original protocol have been discussed with the TSC. All relevant parties, including the ISRCTN, have been notified of changes to the protocol.
Consent or assent	26a	Who will obtain informed consent or assent from potential trial participants or authorised surrogates, and how (see Item 32)<br>- Written informed consent will be obtained by researchers in the trial, as described on page 6-7.
	26b	Additional consent provisions for collection and use of participant data and biological specimens in ancillary studies, if applicable<br>- Not applicable
Confidentiality	27	How personal information about potential and enrolled participants will be collected, shared, and maintained in order to protect confidentiality before, during, and after the trial<br>- Page 12
Declaration of interests	28	Financial and other competing interests for principal investigators for the overall trial and each study site<br>- The authors declare no conflict of interests
Access to data	29	Statement of who will have access to the final trial dataset, and disclosure of contractual agreements that limit such access for investigators<br>- Only the immediate research team will have access to the final trial dataset.
Ancillary and post-trial care	30	Provisions, if any, for ancillary and post-trial care, and for compensation to those who suffer harm from trial participation<br>- There will be no post-trial care for participants who were randomised to treatment as usual as part of the original design of the study. This has been communicated to participants upon enrolling in the trial.
Dissemination policy	31a	Plans for investigators and sponsor to communicate trial results to participants, healthcare professionals, the public, and other relevant groups (eg, via publication, reporting in results databases, or other data sharing arrangements), including any publication restrictions<br>- Page 13

For peer review

For peer review only - http://bmjopen.bmj.com/site/about/guidelines.xhtml

	31b	Authorship eligibility guidelines and any intended use of professional writers<br>- Authorship will follow the ICMJE recommendations (substantial contribution to the design of the study, analysis or data interpretation, drafting or revising the work, final approval or the published version and agreement to be accountable for all aspects of the work).
	31c	Plans, if any, for granting public access to the full protocol, participant-level dataset, and statistical code<br>- The full protocol can be accessed online at https://www.isrctn.com/ISRCTN31976295?q=&filters=&sort=&offset=3&totalResults=16965&page=1&pageSize=10&searchType=basic-search<br>- Participants gave consent for their data to be anonymised and made available for use in future studies. Data will be available upon reasonable request via the first author.
**Appendices**		
Informed consent materials	32	Model consent form and other related documentation given to participants and authorised surrogates<br>- Please see the attached Participant Information Sheet
Biological specimens	33	Plans for collection, laboratory evaluation, and storage of biological specimens for genetic or molecular analysis in the current trial and for future use in ancillary studies, if applicable<br>- Not applicable

*It is strongly recommended that this checklist be read in conjunction with the SPIRIT 2013 Explanation & Elaboration for important clarification on the items. Amendments to the protocol should be tracked and dated. The SPIRIT checklist is copyrighted by the SPIRIT Group under the Creative Commons "Attribution-NonCommercial-NoDerivs 3.0 Unported" license.

For peer review only
